# From insight network to open policy practice: practical experiences

**DOI:** 10.1186/s12961-020-00547-3

**Published:** 2020-04-03

**Authors:** Jouni T. Tuomisto, Mikko V. Pohjola, Teemu J. Rintala

**Affiliations:** 1 Finnish Institute for Health and Welfare, Kuopio, Finland; 2 Kisakallio Sports Institute, Lohja, Finland; 3 Institute of Biomedicine, University of Eastern Finland, Kuopio, Finland

**Keywords:** Environmental health, Decision support, Open assessment, Open policy practice, Shared understanding, Policy-making, Collaboration, Evaluation, Knowledge crystal, Impact assessment

## Abstract

**Background:**

Evidence-informed decision-making and better use of scientific information in societal decisions has been an area of development for decades but is still topical. Decision support work can be viewed from the perspective of information collection, synthesis and flow between decision-makers, experts and stakeholders. Open policy practice is a coherent set of methods for such work. It has been developed and utilised mostly in Finnish and European contexts.

**Methods:**

An overview of open policy practice is given, and theoretical and practical properties are evaluated based on properties of good policy support. The evaluation is based on information from several assessments and research projects developing and applying open policy practice and the authors’ practical experiences. The methods are evaluated against their capability of producing quality of content, applicability and efficiency in policy support as well as how well they support close interaction among participants and understanding of each other’s views.

**Results:**

The evaluation revealed that methods and online tools work as expected, as demonstrated by the assessments and policy support processes conducted. The approach improves the availability of information and especially of relevant details. Experts are ambivalent about the acceptability of openness – it is an important scientific principle, but it goes against many current research and decision-making practices. However, co-creation and openness are megatrends that are changing science, decision-making and the society at large. Against many experts’ fears, open participation has not caused problems in performing high-quality assessments. On the contrary, a key challenge is to motivate and help more experts, decision-makers and citizens to participate and share their views. Many methods within open policy practice have also been widely used in other contexts.

**Conclusions:**

Open policy practice proved to be a useful and coherent set of methods. It guided policy processes toward a more collaborative approach, whose purpose was wider understanding rather than winning a debate. There is potential for merging open policy practice with other open science and open decision process tools. Active facilitation, community building and improving the user-friendliness of the tools were identified as key solutions for improving the usability of the method in the future.

## Background

This article describes and evaluates open policy practice, a set of methods and tools for improving evidence-informed policy-making. Evidence-informed decision support has been a hot and evolving topic for a long time, and its importance is not diminishing any time soon. In this article, decision support is defined as knowledge work that is performed during the complete decision process (ideating possible actions, assessing impacts, deciding between options, implementing decisions and evaluating outcomes) and that aims to produce better decisions and outcomes [1]. Here, ‘assessment of impacts’ means ex ante consideration about what will happen if a particular decision is made, and ‘evaluation of outcomes’ means ex post consideration about what did happen after a decision was implemented.

The area is complex, and the key players — decision-makers, experts and citizens or other stakeholders — all have different views on the process, their own roles in it, and how information should be used in the process. For example, researchers often think of information as a way to find the truth, while politicians see information as one of the tools to promote political agendas ultimately based on values [2]. Therefore, a successful method should provide functionalities for each of the key groups.

In the late 1970s, the focus was on scientific knowledge and an idea that political ambitions should be separated from objective assessments, especially in the United States. Since the 1980s, risk assessment has been a key method to assess human risks of environmental and occupational chemicals [3]. The National Research Council specifically developed a process that could be used by all federal United States agencies. The report emphasised the importance of scientific knowledge in decision-making and scientific methods, such as critical use of data, as integral parts of assessments. Criticism based on observations and rationality is a central idea in the scientific method [4]. The report also clarified the use of causality: the purpose of an assessment is to clarify and quantify a causal path where an exposure to a chemical or other agent leads to a health risk via pathological changes described by the dose–response function of that chemical.

The approach was designed for single chemicals rather than for complex societal issues. This shortcoming was approached in another report that acknowledged this complexity and offered deliberation with stakeholders as a solution, in addition to scientific analysis [5]. An idea was to explicate the intentions of the decision-maker but also those of the public. Additionally, mutual learning about the topic was seen as important. There are models for describing facts and values in a coherent dual system [6]. However, practical assessments have found it difficult to successfully perform deliberation on a routine basis [7]. Indeed, citizens often complain that, even if they have been formally listened to during a process, the processes need more openness as their concerns have not contributed to the decisions made [8].

Western societies have shown a megatrend of increasing openness in many sectors, including decision-making and research. Openness of scientific publishing is increasing and many research funders also demand publishing of data, and research societies are starting to see the publishing of data as a scientific merit in itself [9]. It has been widely acknowledged that the current mainstream of proprietary (as opposed to open access) scientific publishing is a hindrance to spreading ideas and ultimately science [10]. Additionally, governments have been active in opening data and statistics to wide use (data.gov.uk). Governance practices have been developed towards openness and inclusiveness, promoted by international initiatives such as Open Government Partnership (www.opengovpartnership.org).

As an extreme example, a successful hedge fund – Bridgewater Associates – implements radical openness and continuous criticism of all ideas presented by its workers rather than letting organisational status determine who is heard [11]. In a sense, they are implementing the scientific method in much more rigorous way than what is typically done in science.

In the early 2000s, several important books and articles were published about mass collaboration [12], wisdom of crowds [13], crowdsourcing in the government [14] and co-creation [15]. A common idea of the authors was that voluntary, self-organised groups had knowledge and capabilities that could be much more effectively harnessed in the society than what was happening at the time. Large collaborative projects have shown that, in many cases, they are very effective ways to produce high-quality information as long as quality control systems are functional. In software development, the Linux operating system, Git software and the Github platform are examples of this. Additionally, Wikipedia, the largest and most used encyclopaedia in the world, has demonstrated that self-organised groups can indeed produce high-quality content [16].

The five principles of collaboration, openness, causality, criticism and intentionality (Table 1) were seen as potentially important for environmental health assessment in the Finnish Institute for Health and Welfare (at that time the National Public Health Institute), and they were adopted in the methodological decision support work of the Centre of Excellence for Environmental Health Risk Analysis (2002–2007). Open policy practice has been developed over the last 20 years especially to improve environmental health assessments.^1^ Developers have come from several countries in projects mostly funded by the EU and the Academy of Finland (see Funding and Acknowledgements).

**Table 1:** Principles of open policy practice (Collaboration, Openness, Causality, Criticism, Intentionality principles)

Principle	Description
Collaboration	Knowledge work is performed together in aim to produce shared information.
Openness	All work and information are openly available for reading and contributing to anyone interested at all times. If there are exceptions, these must be publicly justified.
Causality	The focus is on understanding and describing the causal relations between the decision options and the intended outcomes. The aim is to predict what impacts will likely occur if a particular decision option is chosen.
Criticism	All information presented can be criticised based on relevance and accordance to observations. The aim is to reject ideas, hypotheses — and ultimately decision options — that do not hold against critique.
Intentionality	The decision-makers explicate their objectives and decision options under consideration. Additionally, values of other participants or stakeholders are documented and considered.

Materials for the development, testing and evaluation of open policy practice were collected from several sources.

Research projects about assessing environmental health risks were an important platform to develop, test and implement assessment methods and policy practices. Important projects are listed in the Funding section. In particular, the Sixth Framework Programme of the EU and its INTARESE and HEIMTSA projects (2005–2011) enabled active international collaboration around environmental health assessment methods.

Assessment cases were performed in research projects and in support for national or municipality decision-making in Finland. Methods and tools were developed side by side with practical assessment work (Additional file 1: Appendix S1).

Literature searches were performed to retrieve scientific and policy literature and websites. Concepts and methods similar to those in open policy practice were sought. Data was searched from PubMed, Web of Knowledge, Google Scholar and the Internet. In addition, a snowball method was used, wherein found documents were used to screen their references and authors of other publications to identify new publications. Herein four articles that describe large literature searches and their results were included [1, 7, 17, 18].

Open risk assessment workshops were organised as spin-offs of several of these projects for international doctoral students in 2007, 2008 and 2009. The workshops offered a place to share, discuss and criticise ideas.

A Master’s course Decision Analysis and Risk Management (6 credit points) was organised by the University of Eastern Finland (previously University of Kuopio) in 2011, 2013, 2015 and 2017. The course taught open policy practice and tested its methods in course work.

Finally, general expertise and understanding was developed during practical experiences and long-term follow-up of international and national politics.

The development and selection of methods and tools to open policy practice has roughly followed an iterative pattern, where an idea is improved during each iteration, or sometimes rejected. The iterative pattern is as follows:

- A need is identified for improving knowledge practices of a decision process or scientific policy support; this need typically arises from scientific literature, project work or news media.
- A solution idea is developed with the aim to tackle the need.
- Whether the idea fits logically in the current framework of open policy practice is then assessed.
- The idea is discussed in a project team to develop it further and gain acceptance.
- A practical solution (web tool, checklist or similar) is produced.
- The solution is piloted in an assessment or policy process.
- The solution is added into the recommended set of methods of open policy practice.
- The method is updated based on practical experience.

Development of open policy practice started with a focus on opening the expert work in policy assessments. In 2007, this line of research produced a summary report about the new methods and tools developed to facilitate assessments [19]. Later, a wider question about open policy practice^2^ emerged – how to organise evidence-informed decision-making in a situation where the five principles are used as the starting point? The question was challenging, especially as it was understood that societal decision-making is rarely a single event, but often consists of several interlinked decisions at different time points and sometimes by several decision-making bodies. Therefore, it was seen more as a leadership guidance rather than as advice about a single decision.

This article gives the first comprehensive, peer-reviewed description about the current methods and tools of open policy practice since the 2007 report [19]. Case studies have been published along the way, and the key methods have been described in different articles. Additionally, all methods and tools have been developed online and the full material has been available at Opasnet (http://en.opasnet.org) for interested readers since each piece was first written.

The purpose of this article is to critically evaluate the performance of open policy practice. Does open policy practice have the properties of good policy support? And, does it enable policy support according to the five principles delineated in Table 1?

## Open policy practice

In this section, open policy practice is described in its current state. First, an overview is given, and then each part is described in more detail.

**Fig. 1:**
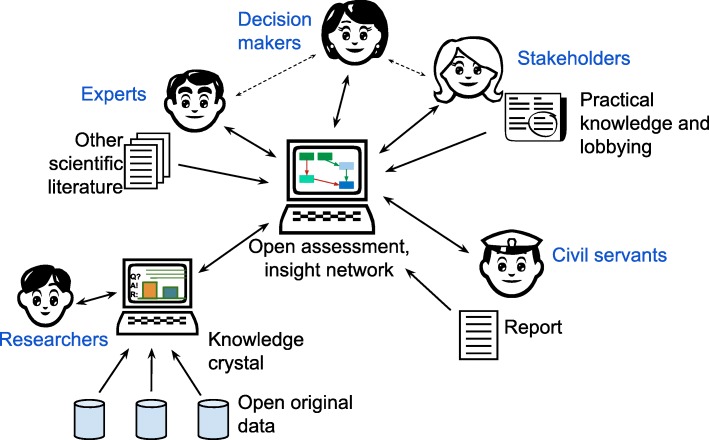
Information flows in open policy practice. Open assessments and web-workspaces have an important role as information hubs. They collect relevant information for particular decision processes and organise and synthesise it into useful formats especially for decision-makers but also for anyone. The information hub works more effectively if all stakeholders contribute to one place or alternatively facilitators collect their contributions there

Open policy practice is a set of methods to support and perform societal decision-making in an open society, and it is the overarching concept covering all methods, tools, practices and terms presented in this article [20]. Its theoretical foundation is on the graph theory [21] and systematic information structures. Open policy practice especially focuses on promoting the openness, flow and use of information in decision processes (Fig. 1). Its purpose is to give practical guidance for the whole decision process from ideating possible actions to assessing impacts, deciding between options, implementing decisions and, finally, to evaluating outcomes. It aims to be applicable to all kinds of societal decision situations in any administrative area or discipline. An ambitious objective of open policy practice is to be so effective that a citizen can observe improvements in decisions and outcomes, and so reliable that a citizen is reluctant to believe claims that are in contradiction with shared understanding produced by open policy practice.

**Fig. 2:**
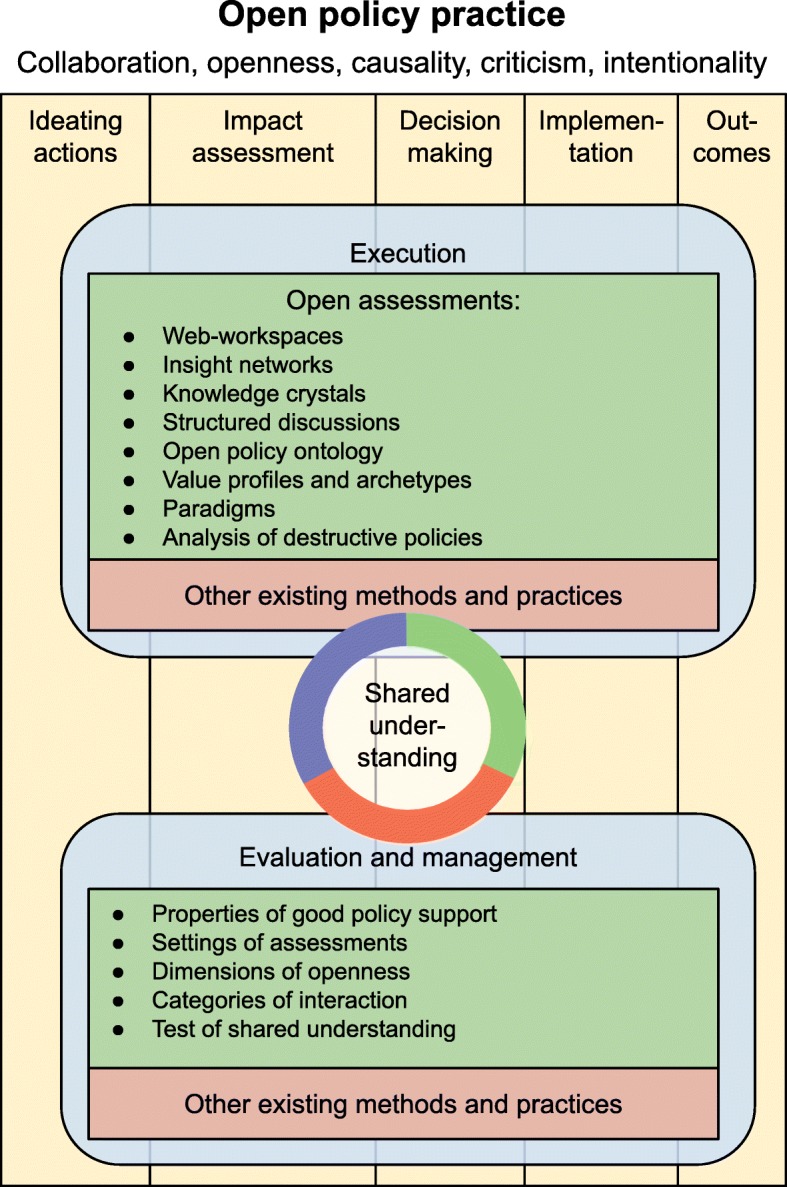
The three parts of open policy practice. The timeline goes roughly from left to right, but all work should be seen as iterative processes. Shared understanding as the main output is in the middle, expert-driven information production is a part of execution. Evaluation and management gives guidance to the execution

Open policy practice is based on the five principles presented in Table 1. The principles can be met if the purpose of policy support is set to produce ‘shared understanding’ (a situation where different facts, values and disagreements related to a decision situation are understood and documented). The description of shared understanding (and consequently improved actions) is thus the main output of open policy practice (Fig. 2). It is a product that guides the decision and is the basis for evaluation of outcomes.

This guidance is formalised as ‘evaluation and management’ of the work and knowledge content during a decision process. It defines the criteria against which the knowledge process needs to be evaluated and managed. It contains methods to look at what is being done, whether the work is producing the intended knowledge and outputs, and what needs to be changed. Each task is evaluated before, during, and after the actual execution, and the work is iteratively managed based on this.

The ‘execution’ of a decision process is about collecting, organising and synthesising scientific knowledge and values in order to achieve objectives by informing the decision-maker and stakeholders. A key part is open assessment that typically estimates the impacts of the planned decision options. Assessment and knowledge production is also performed during the implementation and evaluation steps. Execution also contains the acts of making and implementing decisions; however, they are such case-specific processes depending on the topic, decision-maker and the societal context that they are not discussed in this article.

### Shared understanding

Shared understanding is a situation where all participants’ views about a particular topic have been understood, described and documented well enough so that people can know what facts, opinions, reasonings and values exist, and what agreements and disagreements exist and why. Shared understanding is produced in collaboration by decision-makers, experts and stakeholders. Each group brings in their own knowledge and concerns. Shared understanding aims to reflect all the five principles of open policy practice. This creates requirements to the methods that can be used to produce shared understanding.

Shared understanding is always about a particular topic and produced by a particular group of participants. Depending on the participants, the results might differ, but with an increasing number of participants, it putatively approaches a shared understanding of the society as a whole. Ideally, each participant agrees that the written description correctly contains their own thinking about the topic. Participants should even be able to correctly explain what other thoughts there are and how they differ from their own. Ideally, any participant can learn, understand and explain any thought represented in the group. Importantly, there is no need to agree on things, just to agree on what the disagreements are about. Therefore, shared understanding is not the same as consensus or agreement.

Shared understanding has several potential purposes, all of which aim to improve the quality of societal decisions. It helps people understand complex policy issues and see their own thoughts from a wider perspective and thus increase acceptance of decisions. Additionally, it improves trust in decision-makers, but it may also deteriorate trust if the actions of a decision-maker are not understandable based on shared understanding. It dissects each difficult detail into separate discussions and then collects statements into an overview; this helps to efficiently allocate the time resources of participants to critical issues. Finally, it improves awareness of new ideas and releases the full potential of the public to prepare, inform and make decisions. How well these purposes have been fulfilled in practice in assessments are discussed in the Results section.

### Test of shared understanding

A ‘test of shared understanding’ can be used to evaluate how well shared understanding has been achieved. In a successful case, all participants of a decision process give positive answers to the questions in Table 2. In a way, shared understanding is a metric for evaluating how well decision-makers have embraced the knowledge base of the decision situation.

**Table 2:** Test of shared understanding

Question	Who is asked?
Is all relevant and important information described?	All participants of the decision processes (including knowledge gathering processes)
Are all relevant and important value judgements described? (Those of all participants, not just decision-makers)	
Are the decision-maker’s decision criteria described?	
Is the decision-maker’s rationale from the criteria to the decision described?	

Everything that is done aims to offer better understanding about impacts of the decision related to the decision-maker’s objectives. However, conclusions may be sensitive to initial values, and ignoring stakeholders’ views may cause trouble at a later stage. Therefore, other values in society are also included in shared understanding.

Shared understanding may have different levels of ambition. On an easy level, shared understanding is taken as general guidance and an attitude towards other people’s opinions. Main points and disagreements are summarised in writing, so that an outsider is able to understand the overall picture.

On an ambitious level, the idea of documenting all opinions and their reasonings is taken literally. Participants’ views are actively elicited and tested to see whether a facilitator is able to reproduce their thought processes. The objective here is to document the thinking in such a detailed way that a participant’s views on the key questions of a policy can be anticipated from the description they have given. This is done by using insight networks, knowledge crystals and other methods (see below). Written documentation with an available and usable structure is crucial, as it allows participation without being physically present. It also spreads shared understanding to decision-makers and to those who were not involved in discussions.

Good descriptions of shared understanding are able to quickly and easily incorporate new information or scenarios from the participants. They can be examined using different premises, i.e. a user should be able to quickly update the knowledge base, change the point of view, or reanalyse how the situation would look like with alternative valuations. Ideally, a user interface would allow the user to select input values with intuitive menus and sliders and would show impacts of changes instantly.

Shared understanding as the key objective gives guidance to the policy process in general. However, it also creates requirements that can be described as quality criteria for the process and used to evaluate and manage the work.

### Evaluation and management

‘Evaluation’ is about following and checking the plans and progress of the decisions and implementation. ‘Management’ is about adjusting work and updating actions based on evaluation to ensure that objectives are reached. Several criteria were developed in open policy practice to evaluate and describe the decision support work. Their purpose is to help participants focus on the most important parts of open policy practice. Guidance exists about crowdsourced policy-making [22], and similar ideas have been utilised in open assessment.

### Properties of good policy support

There is a need to evaluate an assessment work before, during and after it is done [17]. A key question is, what makes good policy support and what criteria should be used (Table 3)? [23].

**Table 3:** Properties of good policy support. Here, ‘assessment’ can be viewed as a particular expert work producing a report about a specific question, or as a wider description of shared understanding about a whole policy process; assessment work is done before, during and after the actual decision

Category	Description	Guiding questions	Related principles
Quality of content	Specificity, exactness and correctness of information; correspondence between questions and answers	How exact and specific are the ideas in the assessment? How completely does the (expected) answer address the assessment question? Are all important aspects addressed? Is there something unnecessary?	Openness, causality, criticism
Applicability	*Relevance*: Correspondence between output and its intended use	How well does the assessment address the intended needs of the users? Is the assessment question good in relation to the purpose of the assessment?	Collaboration, openness, criticism, intentionality
	*Availability*: Accessibility of the output to users in terms of, for example, time, location, extent of information, extent of users	Is the information provided by the assessment available when, where and to whom is needed?	Openness
	*Usability*: Potential of the information in the output to generate understanding among its user(s) about the topic of assessment	Are the intended users able to understand what the assessment is about? Is the assessment useful for them?	Collaboration, openness, causality, intentionality
	*Acceptability*: Potential of the output being accepted by its users; fundamentally a matter of its making and delivery, not its information content	Is the assessment (both its expected results and the way the assessment is planned to be made) acceptable to the intended users?	Collaboration, openness, criticism, intentionality
Efficiency	Resource expenditure of producing the assessment output either in one assessment or in a series of assessments	How much effort is needed for making the assessment? Is it worth spending the effort, considering the expected results and their applicability for the intended users? Are the assessment results useful for some other purpose?	Collaboration, openness

Fulfilling all these criteria is of course not a guarantee that the outcomes of a decision will be successful. However, the properties listed have been found to be important determinants of the success of decision processes. In projects utilising open policy practice, poor performance of specific properties could be linked to particular problems observed. Evaluating these properties before or during a decision process could help to analyse what exactly is wrong, as problems with such properties are, by then, typically visible. Thus, using this evaluation scheme proactively makes it possible to manage the decision-making process towards higher quality of content, applicability and efficiency.

‘Quality of content’ refers to the output of an assessment, typically a report, model or summary presentation. Its quality is obviously an important property. If the facts are plain wrong, it is more likely to misguide than lead to good decisions. Specificity, exactness and correctness describe how large the remaining uncertainties are and how close the answers probably are to the truth (compared to some golden standard). In some statistical texts, similar concepts have been called precision and accuracy, although with decision support they should be understood in a flexible rather than strictly statistical sense [24]. Coherence means that the answers given are those to the questions asked.

‘Applicability’ is an important aspect of evaluation. It looks at properties that affect how well the decision support can and will be applied. It is independent of the quality of content, i.e. despite high quality, an assessment may have very poor applicability. The opposite may also be true, as sometimes faulty assessments are actively used to promote policies. However, usability typically decreases rapidly if the target audience evaluates an assessment to be of poor quality.

Relevance asks whether a good question was asked to support decisions. Identification of good questions requires much deliberation between different groups, including decision-makers and experts, and online forums may potentially help in this.

Availability is a more technical property and describes how easily a user can find the information when needed. A typical problem is that a potential user does not know that a piece of information exists even if it could be easily accessed.

Usability may differ from user to user, depending on, for example, background knowledge, interest or time available to learn the content.

Acceptability is a very complex issue and most easily detectable when it fails. A common situation is that stakeholders feel that they have not been properly heard and therefore any output from decision support is perceived as faulty. Doubts about the credibility of the assessor also fall into this category.

‘Efficiency’ evaluates resource use when performing an assessment or other decision support. Money and time are two common measures for this. Often, it is most useful to evaluate efficiency before an assessment is started. Is it realistic to produce new important information given the resources and schedule available? If more/less resources were available, what value would be added/lost? Another aspect in efficiency is that, if assessments are done openly, reuse of information becomes easier and the marginal cost and time of a new assessment decreases.

All properties of decision support, not just efficiency or quality of content, are meant to guide the planning, execution and evaluation of the whole decision support work. If they are always kept in mind, they can improve daily work.

### Settings of assessments

Sometimes, a decision process or an assessment may be missing a clear understanding of what should be done and why. An assessment may even be launched in hope that it will somehow reveal what the objectives or other important factors are. ‘Settings of assessments’ (Table 4) are used to explicate these so that useful decision support can be provided [25]. Examining the sub-attributes of an assessment question can also help the (1) research question – the actual question of an open assessment; (2) boundaries – temporal, geographical and other limits within which the question is considered; (3) decisions and scenarios – decisions and options to assess and scenarios to consider; (4) timing – the schedule of the assessment work; (5) participants – people who will or should contribute to the assessment; and (6) users and intended use – users of the final assessment report and purposes of its use.

**Table 4:** Important settings for environmental health and other impact assessments within the context public policy-making

Attribute	Guiding questions	Example categories
Impacts	• Which impacts are addressed in assessment? • Which impacts are the most significant? • Which impacts are the most relevant for decision-making?	Environment, health, cost, equity
Causes	• Which causes of impacts are recognised in assessment? • Which causes of impacts are the most significant? • Which causes of impacts are the most relevant for decision-making?	Production, consumption, transport, heating, power production, everyday life
Problem owner	• Who has the interest, responsibility and/or means to assess the issue? • Who actually conducts the assessment? • Who has the interest, responsibility and/or power to make decisions and take actions upon the issue? • Who is affected by the impacts?	Policy-maker, industry, business, expert, consumer, public
Target users	• Who is the intended users of assessment results? • Who needs the assessment results? • Who can make use of the assessment results?	Policy-maker, industry, business, expert, consumer, public
Interaction	• What is the degree of openness in assessment (and management)? (see Table 5) • How does assessment interact with the intended use of its results? (see Table 6) • How does assessment interact with other actors in its context?	Isolated, informing, participatory, joint, shared

### Interaction and openness

In open policy practice, the method itself is designed to facilitate openness in all its dimensions. The ‘dimensions of openness’ help to identify if and how the work deviates from the ideal of openness, so that the work can be improved in this respect (Table 5) [18].

**Table 5:** Dimensions of openness in decision-making

Dimension	Description
Scope of participation	Who is allowed to participate in the process?
Access to information	What information about the issue is made available to participants?
Timing of openness	When are participants invited or allowed to participate?
Scope of contribution	Which aspects of the issue are participants invited or allowed to contribute to?
Impact of contribution	How much are participant contributions allowed to have influence on the outcomes? How much weight is given to participant contributions?

Openness can also be examined based on how intensive it is and what kind of collaboration between decision-makers, experts and stakeholders is aimed for [7, 26]. Different approaches are described in Table 6. These evaluation methods guide the actual execution of a decision process.

**Table 6:** Categories of interaction within the knowledge–policy interaction framework

Category	Description
Isolated	Assessment and use of assessment results are strictly separated; results are provided for intended use, but users and stakeholders cannot interfere with the making of the assessment
Informing	Assessments are designed and conducted according to specified needs of intended use; users and limited groups of stakeholders may have a minor role in providing information to the assessment, but mainly serve as recipients of assessment results
Participatory	Broader inclusion of participants is emphasised; participation is, however, treated as an add-on alongside the actual processes of assessment and/or use of assessment results
Joint	Involvement and exchange of summary-level information among multiple actors is emphasised in scoping, management, communication and follow-up of assessment; on the level of assessment practice, actions by different actors in different roles (assessor, manager, stakeholder) remain separate
Shared	Different actors engage in open collaboration upon determining assessment questions, seeking answers to them, and implementing answers in practice; however, the actors involved in an assessment retain their roles and responsibilities

### Execution and open assessment

‘Execution’ is the work during a decision process, including ideating possible actions, assessing impacts, deciding between options, implementing decisions and evaluating outcomes. Execution is guided by information produced in evaluation and management. The focus of this article is on knowledge processes that support decisions. Therefore, methods to reach or implement a decision are not discussed here.

‘Open assessment’ is a method for performing impact assessments using insight networks, knowledge crystals and web-workspaces (see below). Open assessment is an important part of execution and the main knowledge production method in open policy practice.

An assessment aims to quantify important objectives and especially compare differences in impacts resulting from different decision options. In an assessment, current scientific information is used to answer policy-relevant questions that inform decision-makers about the impacts of different options.

Open assessments are typically performed before a decision is made (but, for example, the city of Helsinki has used both ex ante and ex post approaches with its climate strategy [27]). The focus is by necessity on expert knowledge and how to organise it, although prioritisation is only possible if the objectives and valuations of the decision-maker and stakeholders are known. For a list of major open assessments, see Additional file 1: Appendix S1.

As a research topic, open assessment attempts to answer this question: ‘How can factual information and value judgements be organised for improving societal decision-making in a situation where open participation is allowed?’ As can be seen, openness, participation and values are taken as given premises. This was far from common practice. but not completely new, when the first open assessments were performed in the early 2000s [5].

Since the beginning, the main focus has been to think about information and information flows, rather than jurisdictions, political processes or hierarchies. Therefore, open assessment deliberately focuses on impacts and objectives rather than questions about procedures or mandates of decision support. The premise is that, if the information production and dissemination are completely open, the process can be generic, and an assessment can include information from any contributor and inform any kind of decision-making body. Of course, quality control procedures and many other issues must be functional under these conditions.

### Co-creation

‘Co-creation’ is a method for producing open contents in collaboration, and in this context specifically knowledge production by self-organised groups. It is a discipline in itself [15], and guidance about how to manage and facilitate co-creation can be found elsewhere. Here, only a few key points are raised about facilitation and structured discussion.

Information has to be collected, organised and synthesised; facilitators need to motivate and help people to share their information. This requires dedicated work and skills that are not typically available among experts nor decision-makers. Co-creation also contains practices and methods, such as motivating participation, facilitating discussions, clarifying and organising argumentation, moderating contents, using probabilities and expert judgement for describing uncertainties, or developing insight networks (see below) or quantitative models. Sometimes, the skills needed are called ‘interactional expertise’.

Facilitation helps people participate and interact in co-creation processes using hearings, workshops, online questionnaires, wikis, and other tools. In addition to practical tools, facilitation implements principles that have been seen to motivate participation [14]. Three are worth mentioning here because they have been shown to significantly affect the motivation to participate, as follows: (1) grouping – facilitation methods are used to promote the participants’ feeling of being important members of a group that has a meaningful, shared purpose; (2) trust – facilitation builds trust among people that they can safely express their ideas and concerns, and that other members of the group support participation even if they disagree on the substance; and (3) respect – contributions are systematically evaluated according to their merit so that each participant receives the respect they deserve based on their contributions as individuals or members of a group.

‘Structured discussions’ are synthesised and reorganised discussions, where the purpose is to highlight key statements, and argumentations that lead to acceptance or rejection of these statements. Discussions can be organised according to pragma-dialectical argumentation rules [28] or argumentation framework [29], so that arguments form a hierarchical thread point to a main statement or statements. Attack arguments are used to invalidate other arguments by showing that they are either untrue or irrelevant in their context, defend arguments are used to protect from attacks, and comments are used to clarify issues. For an example, see Figure S2-5 in Additional file 1: Appendix S2 and links thereof.

The discussions can be natural discussions that are reorganised afterwards or online discussions where the structure of contributions is governed by the tools used. A test environment exists for structured argumentation [30], and Opasnet has R functions for analysing structured discussions written on wiki pages.

### Insight networks

**Fig. 3:**
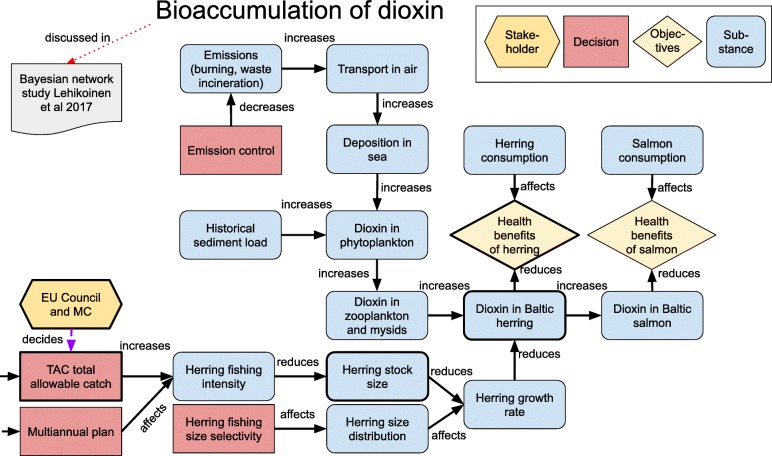
Insight network about dioxins, Baltic fish and health as described in the BONUS GOHERR project [31]. Decisions are shown as red rectangles, decision-makers and stakeholders as yellow hexagons, decision objectives as yellow diamonds, and substantive issues as blue nodes. The relations are written on the diagram as predicates of sentences where the subject is at the tail of the arrow and the object is at the tip of the arrow. For other insight networks, see Additional file 1: Appendix S2

‘Insight networks’ are graphs as defined by the graph theory [21]. In an insight network, actions, objectives and other issues are depicted with nodes, and their causal and other relations are depicted with arrows (aka edges). An example is shown in Fig. 3, which describes a potential dioxin-related decision to clean up emissions from waste incineration. The logic of such a decision can be described as a chain or network of causally dependent issues – reduced dioxin emissions to air improve air quality and dioxin deposition into the Baltic Sea; this has a favourable effect on concentrations in the Baltic herring; this reduces human exposures to dioxins via fish; and this helps to achieve an ultimate objective of reduced health risks from dioxin. Insight networks aim to facilitate the understanding, analysis and discussion of complex policy issues.

Causal modelling and causal graphs, as such, are old ideas, and there are various methods developed for them, both qualitative and quantitative. However, the additional ideas with insight networks were that (1) all non-causal issues can and should be linked to the causal core in some way, if they are relevant to the decision, and therefore (2) they can be effectively used in clarifying one’s ideas, and in contributing and then communicating a whole decision situation rather than just the causal core. In other words, a participant in a policy discussion should be able to make a reasonable connection between what they are saying and some node in an insight network developed for that policy issue. If they are not able to make such a link, their point is probably irrelevant.

The first implementations of insight networks were about the toxicology of dioxins [32] and restoration of a closed asbestos mine area [33].^3^ In the early cases, the main purpose was to give structure to discussion about and examination of an issue rather than to be a backbone for quantitative models. In later implementations, such as in the composite traffic assessment [34] or BONUS GOHERR project [31], diagrams have been used for both purposes. Most open assessments discussed later (and listed in Additional file 1: Appendix S1) have used insight networks to structure and illustrate their content.

### Knowledge crystals

‘Knowledge crystals’ are web pages where specific research questions are collaboratively answered by producing rationale with any data, facts, values, reasoning, discussion, models or other information that is needed to convince a critical, rational reader (Table 7).

**Table 7:** The ‘attributes’ of a knowledge crystal

Attribute	Description
Name	An identifier for the knowledge crystal; each page has a permanent, unique name and identifier or URL
Question	A research question that is to be answered; it defines the scope of the knowledge crystal Assessments have specific sub-attributes for questions (see section Settings of assessments)
Answer	An understandable and useful answer to the question; it is the current best synthesis of all available data; typically, it has a descriptive easy-to-read summary and a detailed quantitative ‘result’ published as open data; an answer may contain several competing hypotheses, if they all hold against scientific critique; in this way, it may include an accurate description of the uncertainty of the answer, often in a probabilistic way
Rationale	Any information that is necessary to convince a critical rational reader that the answer is credible and usable; it presents to a reader the information required to derive the answer and explains how it is formed; it may have different sub-attributes depending on the page type, some examples are listed below • Data tell about direct observations (or expert judgements) about the topic. • Dependencies tell what is known about how upstream knowledge crystals (i.e. causal parents) affect the answer; dependencies may describe functional or probabilistic relationships; in an insight network, dependencies are described as arrows pointing toward the knowledge crystal • Calculations are an operationalisation of how to calculate or derive the answer; it uses algebra, computer code, or other explicit methods if possible • Discussions are structured or unstructured discussions about the details of the substance, or about the production of substantive information; on a wiki, discussions are typically located on the talk page of the substance page
Other	In addition to attributes, it is practical to have clarifying subheadings on a knowledge crystal page; these include self-explanatory subheadings such as See also, Keywords, References, Related files

Knowledge crystals have a few distinct features. The web page of a knowledge crystal has a permanent identifier or URL and an explicit topic, or question, which does not change over time. A user may come to the same page several times and find an up-to-date answer to the same topic. The answer changes as new information becomes available, and anyone is allowed to bring in new relevant information as long as certain rules of co-creation are followed. In a sense, the answer of a knowledge crystal is never final but it is always usable.

Knowledge crystals are a practical information structure designed to comply with the principles of open policy practice. Open data principles are used when possible [35]. For example, openness and criticism are implemented by allowing anyone to contribute but only after critical examination. Knowledge crystals differ from open data, which contains little to no interpretation, and scientific articles, which are not updated. Their rationale is a place for new information and discussions, and resolutions about new information may change the answer.

The purpose of knowledge crystals is to offer a versatile information structure for nodes in an insight network that describes a complex policy issue. They handle research questions of any topic and describe all causal and non-causal relations from other nodes (i.e. the nodes that may affect the answer of the node under scrutiny). They contain information as necessary – text, images, mathematics or other forms, both quantitative and qualitative. They handle facts or values depending on the questions and withstand misconceptions and fuzzy thinking as well. Finally, they are intended to be found online by anyone interested, and their main message is intended to be understood and used even by a non-expert.

There are different types of knowledge crystals for different uses. ‘Variables’ contain substantive topics such as emissions of a pollutant, food consumption or other behaviour of an individual, or disease burden in a population (for examples, see Fig. 3 and Additional file 1: Appendix S2). ‘Assessments’ describe the information needs of particular decision situations and work processes designed to answer those needs. They may also describe whole models (consisting of variables) for simulating impacts of a decision. ‘Methods’ describe specific procedures to organise or analyse information. The question of a method typically starts with ‘How to…’. For a list of all knowledge crystal types used at Opasnet web-workspace, see Additional file 1: Appendix S3.

Openness and collaboration are promoted by design – knowledge crystals are modular, re-usable, and readable for humans and machines. This enables their direct use in several assessment models or internet applications, which is important for the efficiency of the work. Methods are used to standardise and facilitate the work across assessments.

### Open web-workspaces

Insight networks, knowledge crystals and open assessments are information objects that were not directly applicable at any web-workspace available at the time of development. Therefore, web-workspaces have been developed specifically for open policy practice. There are two major web-workspaces for this purpose – Opasnet (designed for expert-driven open assessments) and Climate Watch (designed for evaluation and management of climate mitigation policies).

### Opasnet

Opasnet is an open wiki-based web-workspace and prototype for performing open policy practice, launched in 2006. It is designed to offer functionalities and tools for performing open assessments so that most, if not all, work can be done openly online. Its name is a short version of Open Assessors’ Network and also from Finnish word for guide, ‘opas’. The purpose was to test and learn co-creation among environmental health experts and start opening the assessment process to interested stakeholders.

Opasnet is based on the MediaWiki platform because of its open-source code, wide use and abundance of additional packages, long-term prospects, functionalities for good research practices (e.g. talk pages for meta-level discussions), and full and automatic version control. Two language versions of Opasnet exist. English Opasnet (en.opasnet.org) contains all international projects and most scientific information. Finnish Opasnet (fi.opasnet.org) contains mostly project material for Finnish projects and pages targeted for Finnish audiences. A project wiki Heande (short for Health, the Environment, and Everything) requires a password and contains information that cannot (yet) be published, but the open alternatives are preferred.

Opasnet facilitates the simultaneous development of theoretical frameworks, assessment practices, assessment work, and supporting tools. This includes, for example, information structures, assessment methods, evaluation criteria, and online software models and libraries.

For modelling functionalities, the statistical software R is used via an R–Mediawiki interface. R code can be written directly to a wiki page and run by clicking a button. The resulting objects can be stored to the server and fetched later by a different code. Complex models can be run with a web browser without installing anything. The server has automatic version control and archival of the model description, data, code and results.

An R package *OpasnetUtils* is available (CRAN repository cran.r-project.org) to support knowledge crystals and impact assessment models. It contains the necessary functions and information structures. Specific functionalities facilitate reuse and explicit quantitation of uncertainties – scenarios can be defined at a wiki page or via a model user interface, and these scenarios can then be run without changing the model code. If input values are uncertain, uncertainties are automatically propagated through the model using Monte Carlo simulation.

For data storage, Opasnet Base, a MongoDB no-sql database, is used. Each dataset must be linked to a single wiki page, which contains all the necessary descriptions and metadata about the data. Data can be uploaded to the database via a wiki page or a file uploader. The database has an open application programming interface for data retrieval.

For more details, see Additional file 1: Appendix S4.

### Climate Watch

**Fig. 4:**
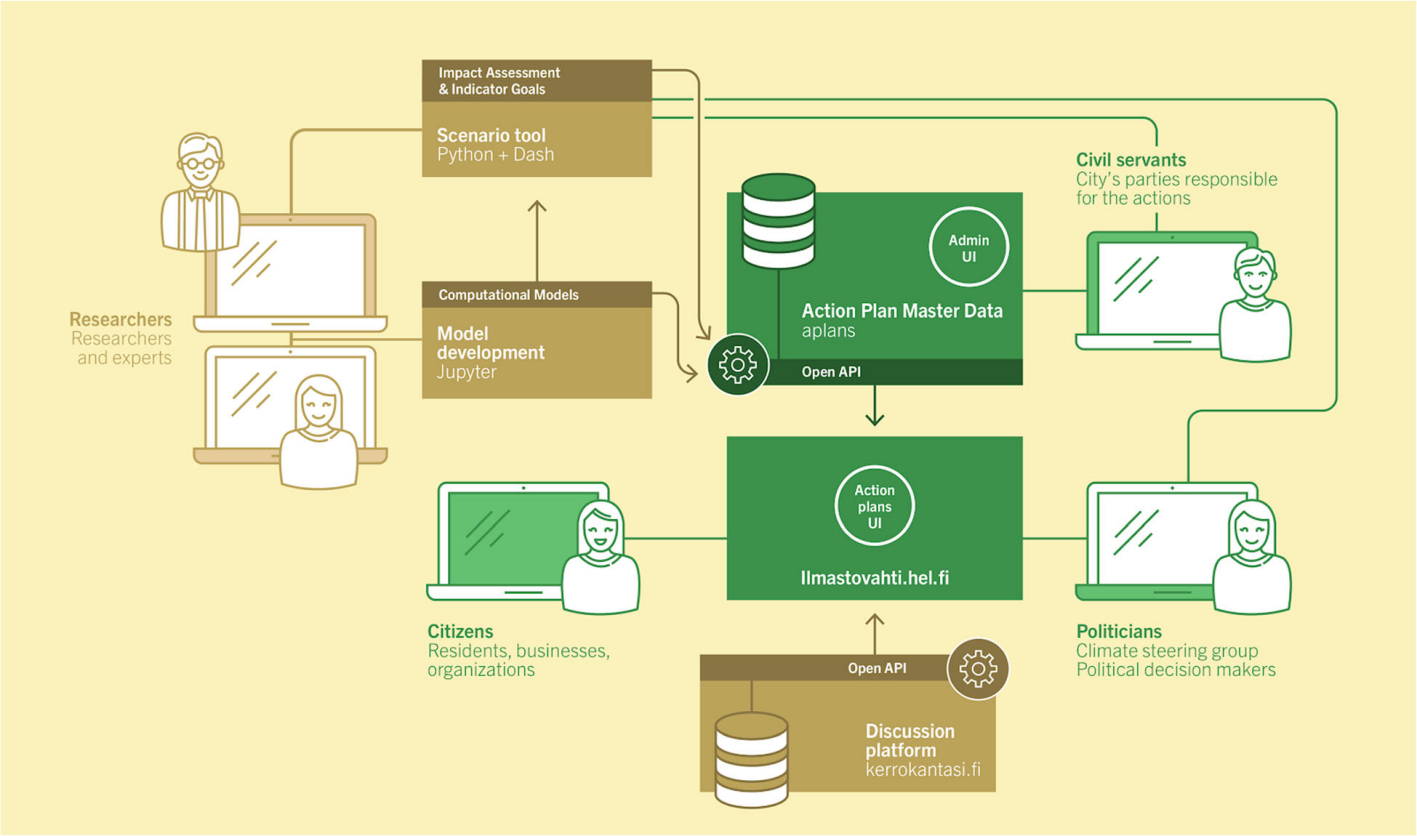
System architecture of the Climate Watch web-workspace

Climate Watch is a web-workspace primarily for evaluating and managing climate mitigation actions (Fig. 4). It was originally developed in 2018–2019 by the city of Helsinki for its climate strategy. Already from the beginning, scalability was a key priority – the web-workspace was made generic enough so that it could be easily used by other municipalities in Finland and globally, and used for evaluation and management of topics other than climate mitigation.

Climate Watch is described in more detail by Ignatius et al. [36]. In brief, Climate Watch consists of actions that aim to reduce climate emissions, and indicators that are supposedly affected by the actions and give insights about progress. Actions and indicators are knowledge crystals, and they are causally connected, thus forming an insight network. Each action and indicator has one or more contact people who are responsible for the reporting of progress (and sometimes for actually implementing the actions).

The requirements for choosing the technologies were wide availability, ease of development, and an architecture based on open application programming interfaces or APIs. The public-facing user interface uses the NextJS framework (https://nextjs.org/). It provides support for server-side rendering and search engine optimisation, which is based on the React user interface framework (https://reactjs.org/). The backend is built using the Django web framework (https://www.djangoproject.com/), which provides the contact people with an administrator user interface. The data flows to the Climate Watch interface over a GraphQL API (https://graphql.org/). GraphQL is a standard that has the most traction in the web development community because of its flexibility and performance.

Opasnet and Climate Watch have functional similarities but different technical solutions. The user interfaces for end-users and administrators in Climate Watch have similar purposes as MediaWiki in Opasnet, and while impact assessment and model development are performed by using R at Opasnet, Climate Watch uses Python, Dash and Jupyter.

### Open policy ontology

‘Open policy ontology’ is used to describe all the information structures and policy content in a systematic, coherent and unambiguous way. The ontology is based on the concepts of open linked data and resource description framework by the World Wide Web Consortium [37].

The ontology is based on vocabularies with specified terms and meanings. Additionally, the relations of terms are explicit. Resource description framework is based on the idea of triples, which have three parts – subject, predicate (or relation) and object. These can be thought as sentences – an item (subject) is related to (predicate) another item or value (object), thus forming a claim. Claims can further be specified using qualifiers and backed up by references. Insight networks can be documented as triples, and a set of triples using this ontology can be visualised as diagrams of insight network. Triple databases enable wide, decentralised linking of various sources and information.

Open policy ontology (Additional file 1: Appendix S3) describes all information objects and terms described above, making sure that there is a relevant item type or relation to every critical piece of information that is described in an insight network, open assessment or shared understanding. A ‘critical piece of information’ means something that is worth describing as a separate node, so that it can be more easily found, understood and used. A node itself may contain large amounts of information and data, but for the purpose of producing shared understanding about a particular decision, there is no need to highlight the node’s internal data on an insight network.

The ontology was used with indicator production in the climate strategy of Helsinki [27] and a visualisation project of insight networks [38].

For a full description of the current vocabulary in the ontology, see Additional file 1: Appendix S3 and Figures S2-3 and S2-4 in Additional file 1: Appendix S2.

### Novel concepts

This section presents novel concepts that have been identified as useful for a particular need and conceptually coherent with open policy practice. However, they have not been thoroughly tested in practical assessments of policy support.

‘Value profile’ is a documented list of values, preferences and choices of a participant. Voting advice applications are online tools that ask electoral candidates about their values, world views or decisions they would make if elected. The voters can then answer the same questions and analyse which candidates share their values. Nowadays, such applications are routinely developed by all major media houses for every national election in Finland. Thus, voting advice applications produce a kind of value profile. However, these tools are not used to collect value profiles from the public for actual decision-making or between elections, although such information could be used in decision support. Value profiles are ‘mydata’, i.e. data of an individual where they themselves can decide who is able to see and use it; this requires trusted and secure information systems.

‘Archetype’ is an internally coherent value profile of an anonymised group of people. Coherence means that, when two values are in conflict, the value profile describes which one to prefer. Archetypes are published as open data describing the number of supporters but not their identities. People may support an archetype in full or by declaring partial support to some specific values. Archetypes aim to save effort in gathering value data from the public, as when archetypes are used, not everyone needs to answer all possible questions. It also increases security since there is no need to handle individual people’s potentially sensitive value profiles, when open aggregated data about archetypes suffices.

Political strategy papers typically contain explicit values of that organisation, aggregated in some way from their members’ individual values. The strategic values are then used in the organisation in a normative way, implying that the members should support these values in their membership roles. An archetype differs from this, because it is descriptive rather than normative and a ‘membership’ in an archetype does not imply any rights or responsibilities. Yet, political parties could also use archetypes to describe the values of their members.

The use of archetypes is based on an assumption that, although their potential number is very large, most of a population’s values relevant for a particular policy can be covered with a manageable amount of archetypes. As a comparison, there are usually from two to a dozen significant political parties in a democratic country rather than hundreds. There is also research on human values showing that they can be systematically evaluated using a fairly small amount (e.g. 4, 10 or 19) of different dimensions [39].

Paradigms are collections of rules to describe inferences that participants would make from data in the system. For example, scientific paradigm has rules about criticism and a requirement that statements must be backed up by data or references. Participants are free to develop paradigms with any rules of their choosing, as long as they can be documented and operationalised within the system. For example, a paradigm may state that, when in conflict, priority is given to the opinion presented by a particular authority. Hybrid paradigms are also allowed. For example, a political party may follow the scientific paradigm in most cases but, when economic assessments are ambiguous, the party chooses an interpretation that emphasises the importance of an economically active state (or, alternatively, market approach with a passive state).

Destructive policy is a policy that (1) is actually being implemented or planned, making it politically relevant, (2) causes significant harm to most or all stakeholder groups, as measured using their own interests and objectives, and (3) has a feasible, less harmful alternative. Societal benefits are likely to be greater if a destructive policy is identified and abandoned, compared with a situation where an assessment only focuses on showing that one good policy option is slightly better than another.

There are a few mechanisms that may explain why destructive policies exist. First, a powerful group can dominate the policy-making to their own benefit, causing harm to others. Second, the ‘prisoner’s dilemma’ or ‘tragedy of commons’ makes a globally optimal solution to be suboptimal for each stakeholder group, thus draining support from it. Third, the issue is so complex that the stability of the whole system is threatened by changes [40]. Advice about destructive policies may produce support for paths out of these frozen situations.

An analysis of destructive policies attempts to systematically analyse policy options and identify, describe, and motivate rejection of those that appear destructive. The tentative questions for such an analysis include the following: (1) Are there relevant policy options or practices that are not being assessed? (2) Do the policy options have externalities that are not being assessed? (3) Are there relevant priorities among stakeholders that are not being assessed? (4) Is there strong opposition against some options among the experts or stakeholders? What is the reasoning for and science behind the opposition? (5) Is there scientific evidence that an option is unable to reach the objectives or is it significantly worse than another option?

The current political actions to mitigate the climate crisis are so far from the global sustainability goals that there must be some destructive policies in place. Identification of destructive policies often requires that an assessor looks out of the box and is not restricted to default research questions. In this example, such questions could be: ‘What is such a policy B that fulfils the objectives of the current policy A but with less climate emissions?’ and ‘Can we reject the null hypothesis that A is better than B in the light of data and all major archetypes?’ This approach has a premise that rejection is more effective than confirmation; an idea that was already presented by Karl Popper [4].

Parts of open policy practice have been used in several assessments. In this article, we will evaluate how these methods have performed.

## Methods

The methods of open policy practice were critically evaluated. The open assessments performed (Additional file 1: Appendix S1) were used as the material for evaluation. The properties of good policy support (Table 3) were used as evaluation criteria in a similar way as in a previous evaluation [23]. In addition, open policy practice as a whole was evaluated using the categories of interaction (Table 6) and the test of shared understanding (Table 2) as criteria [25]. Key questions in the evaluations were the following: Does open policy practice have the properties of good policy support? And does it enable policy support according to the five principles of open policy practice in Table 1? For each method within open policy practice, these questions were asked: In what way could the method materialise improvements in the property considered? Are there evidence or experiences showing that improvement has actually happened in practice? Has the method shown disadvantages or side effects when implemented?

## Results

Different methods of open policy practice were evaluated for their potential or observed advantages and disadvantages according to the properties of good policy support. Major advantages are listed on Table 8. Some advantages as well as disadvantages and problems are discussed in more detail in the text. The text is organised along the properties of good policy support, categories of interaction and test of shared understanding.

**Table 8:** Methods evaluated based on properties of good policy support

Method	Quality of content	Relevance	Availability	Usability	Acceptability	Efficiency
Co-creation	+ Participants bring new info (2, 3, 25, 26)	+ Additional file 1: New questions are identified during collaborative work (6, 11)	+ Draft results raise awareness during work (2, 8, 27)	? Readers ask clarifying questions and learn and create understanding through collaboration	+ Participants are committed to conclusions (2, 8, 27)	? Collaboration integrates communication to decision-makers and stakeholders (users) into the making, which saves time and effort
Open assessment	+ It combines functionalities of other methods and enables peer-reviewed assessment models (4, 5, 16)	+ End-user discussions improve assessment (16, 26, 27)	? It is available as draft since the beginning	+ Standard structure facilitates use (8, 9)	+ Openness was praised (3, 8, 9, 21)	+ Scope can be widened incrementally (12–16)
Insight network	+ It brings structure to assessment and helps causal reasoning (8, 9, 11, 16, 17)	+ It helps and clarifies discussions between decision-makers and experts (8, 9)	-	? Readers see what is excluded	? It helps to check whether important issues are missing	-
Knowledge crystal	+ They streamline work and provide tools for quantitative assessments (e.g. 3, 23, 24)	+ They clarify questions (1, 6)	? It is mostly easy to see where information should be found	? Summaries help to understand	? They make the intentionality visible by describing the assessment question	+ Answers can be reused across assessments (12–16, 23–24)
Web-workspace	+ Its structure supports high-quality content production when moderated (8, 9)	+ It combines user needs and open policy practice (8, 9)	+ It offers an easy approach to and archive of materials (16, 21, 23, 26)	+ The user needs guided the functions developed (8)	-	? It offers a place to document shared understanding and distribute information broadly
Structured discussion	+ It helps to moderate discussion and discourages low-quality contributions (2, 30)	+ It guides focus on important topics (16, 30)	-	? Threads help to focus reading	+ User feedback has been positive: it helps to focus on key issues (8, 30)	? Structure discourages redundancy
Open policy ontology	-	+ It gives structure to insight networks and structured discussions (8, 16, 30)	-	? Ontology clarifies issues and relations	-	-
Value profile and archetype	-	+ Value profiles help to prioritise (8)	-	? Voting advice applications may offer an example	? Stakeholders’ values are better heard	? Archetypes are effective summaries
Paradigm	? It motivates clear reasoning	? It systematically describes conflicting reasonings	-	-	? Stakeholders’ reasonings are better heard	? It helps to analyse inferences of different groups
Analysis of destructive policies	-	+ It widens the scope (3, 8)	-	? It emphasises mistakes to be avoided	? Focus is on everyone’s problems	? Lessons learned can be reused in other decisions
Suggestions by open policy practice	Work openly, invite criticism; use tools and moderation to encourage high-quality contributions (Table 1)	Acknowledge the need for and potential of co-creation, discussion, and revised scoping; invite all to policy-support work; characterize the setting (Table 4)	Design processes and information to be open from the beginning; use open web-workspaces (Table 5)	Invite participation from the problem owner and user groups early on; use user feedback to visualise, clarify and target content (Table 6)	Be open; clarify reasoning; acknowledge disagreements; use the test of shared understanding (Table 2)	Combine information production, synthesis and use to a co-creation process to save time and resources; use shared information objects with open license, e.g. knowledge crystals

In each cell, actual benefit observed in open policy practice materials is marked with '+'. Potential benefit is marked with '?'. No anticipated benefit is marked with '-'. Numbers in parentheses refer to the assessments in Additional file 1: Appendix S1, Table S1-1. The last row contains general suggestions to improve policy support with respect to each property

### Quality of content

Open policy practice aims at high-quality information for decision-makers. One of the ideas is that openness and co-creation enable external experts to see and criticise the content at all times so that corrections can be made. Participation among decision-makers, stakeholders and experts outside an assessment team is typically less common than ideal and requires special effort. The participation has been remarkably higher in projects where special emphasis and effort has been put to dissemination and facilitation such as the Climate Watch and the Transport and Communications Strategy (assessments 8 and 26 in Table S1-1). Resources should be allocated to facilitation already when planning a policy process to ensure useful co-creation.

Participation is a challenge also in Wikipedia, where only a few percent of readers ever contribute and the fraction of active contributors is even smaller [41]. Indeed, the quality of content in Wikipedia is better in topics that are popular and have a lot of contributors.

Active participation did not solve quality control on behalf of the assessors, and it had to be taken care of by usual means. In any case, open policy practice does not restrict the use of common quality control methods and therefore it has at least the same potential to produce high-quality assessments as those using the common methods. The quality of open assessments has been acceptable for publishing in peer-reviewed scientific journals.

### Relevance

What is relevant for a decision process can be a highly disputed topic. The shared interaction implies that stakeholders can and should participate in discussions about relevance and revision of scoping when necessary. In other words, everyone is invited to policy-support work. The setting of an assessment (Table 4) helps participants to see what the assessment is about.

The analysis of destructive policies can be used as a method to focus on critical aspects of an assessment and thus increase relevance. For example, Climate Watch has an impact assessment tool [42] that dynamically simulates the total greenhouse gas emissions of Helsinki based on scenarios provided by the user. The tool is able to demonstrate destructive policies; for example, if the emission factor of district heating production does not significantly decrease in 10 years, it will be impossible to reach the emission targets of Helsinki. Thus, there are sets of solutions that could be chosen because of their appealing details but that would not reduce the emission factor. The tool explicitly demonstrates that these solutions fail to reach the objectives. It also demonstrates that the emission factor is a critical variable that must be evaluated and managed carefully to avoid destructive outcomes.

Other examples include the Helsinki energy decision assessment (assessment 3 in Table S1-1). It showed that residential wood combustion was a devastating way to heat houses in urban areas and health risks were much larger than with any other heating method. Yet, this is a popular practice in Finland, and there is clearly a need for dissemination about this destructive practice. Additionally, a health benefit–risk assessment showed that whatever policy is chosen with dioxins and young women, it should not reduce Baltic fish consumption in other population subgroups (assessment 16 in Table S1-1). This is because the dioxin health risk, while small, is concentrated in the population subgroup of young women, while all other subgroups would clearly benefit from increased fish intake.

### Availability

The tools and web-workspaces presented in this article facilitated the availability of information. In addition, many policy processes were designed in such a way that information was open from the beginning. Increased openness in society has increased demands to make information available in situations where experts used to keep details to themselves. For example, source codes of assessment models have increasingly been made openly available, and Opasnet made that possible for these assessments.

The timing of availability is critical in a policy process, and assessment results are preferably available early on. This is a major challenge, because political processes may proceed rapidly and change focus, and quantitative assessments take time. A positive example of agility was a dioxin assessment model that had been developed in several projects during a few years (assessment 16 in Table S1-1) [31]. When the European Food Safety Authority released their new estimates about dioxin impacts on sperm concentration [43], the assessment model was updated and new sperm concentration results were produced within days. This was possible because the existing dioxin model was modular and using knowledge crystals, so it was rerun after updates in just one part about sperm effects.

The availability of previous versions may be critical. Many experts were reluctant to make their texts available in draft assessments if other people were able edit them, but this fear was often alleviated by the fact that previous versions were always available if needed in Opasnet version control. Availability was also improved as information was produced in a proper format for archiving, backups were produced automatically, and it was easy to produce a snapshot of a final assessment. It was not necessary to copy information from one repository to another, but in a few cases, the final assessments were stored in external open data repositories.

In structured discussion, hierarchical threads increased availability because a reader did not need to read further if they agreed with the topmost arguments (assessment 30 in Table S1-1). On the other hand, any thread could be individually scrutinised to the last detail if needed.

### Usability

Co-creation activities demonstrated the utility of participation and feedback (assessments 6, 8, Table S1-1). Even with good substance knowledge, an assessor cannot know the aspects and concerns a decision-maker may have. The usability of information was clearly improved when problem owners and user groups were invited to participate early on. User feedback proved to be very useful to visualise, clarify and target content.

The climate strategy of Helsinki (assessment 8, Table S1-1) took the usability challenge seriously and developed the Climate Watch website from scratch based on open source code modules and intensive user testing and service design. Insight networks and knowledge crystals were basic building blocks of the system architecture. It received almost exclusively positive feedback from both users and experts. Additionally, a lot of emphasis was put on building a user community and city authorities, other municipalities, and research institutes have shown interest in collaboration. In contrast, Opasnet was designed as generic tool for all kinds of assessments but without an existing end-user demand. As a result, the penetration of Climate Watch has been much quicker.

Insight network provides a method to illustrate and analyse a complex decision situation, while knowledge crystals offer help in describing quantitative nuances within the nodes or arrows such as functional or probabilistic relations or estimates. There are tools with both graphical and modelling functionalities, e.g. Hugin (Hugin Expert A/S, Aalborg, Denmark) for Bayesian belief networks and Analytica® (Lumina Decision Systems Inc, Los Gatos, CA, USA) for Monte Carlo simulation. However, these tools are designed for a single desktop user rather than for open co-creation. In addition, they have limited possibilities for adding non-causal nodes and links or free-format discussions about the topics.

Insight networks were often complex and therefore better suited for detailed expert or policy work rather than for general dissemination. Other dissemination methods were needed as well. This was true also for knowledge crystals, although page summaries helped dissemination.

A knowledge crystal is typically structured so that it starts with a summary, then describes a research question and gives a more detailed answer, and finally provides a user with relevant and increasingly detailed information in a rationale. This increased the usability of a page among different user groups. On the other hand, some people found this structure confusing as they did not expect to see all the details of an assessment. Users were unsure about the status of a knowledge crystal page and whether some information was up to date or still missing. This was because many pages were a work in progress rather than finalised products. This was clarified by adding status declarations on the tops of pages. Declaring drafts as drafts also helped experts who were uncomfortable in showing their own work before it was fully complete.

Voting advice applications share properties with value profiles and archetypes, and offer material for concept development. The popularity of these applications implies that there is a societal need for value analysis and aggregation. The data has been used to understand differences between individuals and political groups in Finland. With more nuanced data, a set of archetypes can probably be developed to describe common and important values in the population. Some of them may have the potential to increase in popularity and form a kind of virtual party that represents a population’s key values.

Value profiles and paradigms were tested on structured discussions and shared understanding descriptions (assessment 30, Table S1-1). Additionally, Helsinki tested value profiles in prioritising the development of Climate Watch. They were found to be promising and conceptually sound ideas in this context. Data that resembles value profiles are being collected by social media companies, but the data are used to inform marketing actions, often without the individual’s awareness, so they are not ‘mydata’. In contrast, the purpose of value profile data is to inform societal decisions with consent from its owner rather than nudge the voter to act according to a social media company’s wishes. The recent microtargeting activities by Cambridge Analytica and AggregateIQ to use value-profile-like data proved to be very effective in influencing voting decisions [44]. Value profiles are clearly severely underutilised as a tool to inform decisions. We are not aware of systems that would collect value profile data for actual democratic policy support between elections.

### Acceptability

A major factor increasing acceptability was whether the stakeholders thought that they had been given all relevant information and whether their concerns had been heard. This emphasised the need to be open and clarify reasonings of different stakeholders. It was also found important to acknowledge disagreements. The test of shared understanding (Table 2) appeared to be a useful tool in documenting these aspects.

Experts were often reluctant to participate in open assessments because they had concerns about the acceptability of the process. They thought that expertise is not given proper weight, if open participation is allowed. They feared that strong lobbying groups hijack the process. They feared that self-organised groups produce low-quality information or even malevolent dis-information. They often demanded the final say as the ultimate quality criteria, rather than trusting that data, reasoning and critical discussion would do a better job. In brief, experts commonly thought that it is simply easier and more efficient to produce high-quality information in closed expert groups.

In a vaccine-related assessment (Additional file 1: Appendix S1, Table S1-1), comments and critique were received from both the drug industry and vaccine citizen organisations by using active facilitation, and they were all very matter-of-fact. This was interesting, as the same topics caused outrage in social media, but this was not seen on structured assessments. This was possibly because the questions asked were specific and typically required some background knowledge of the topic. Interestingly, one of the most common objections and fears against open assessment was that citizen contributions are ill-informed and malevolent. The experience with open assessments showed that they were not.

### Efficiency

Open policy practice combines information production, synthesis and use to a single co-creation endeavour covering a whole policy process. When successful, this approach saved time and resources because of parallel work and rapid feedback and guidance. However, not all open assessments were optimally designed to maximise co-creation between decision-makers and experts. Rather, efficiency was typically achieved when knowledge crystals improved the structure and reuse and thus saved resources in assessment modelling.

A common solution to co-operation needs seemed to be a strict division of tasks. Detailed understanding of and contributions to other groups’ work and models remained low or non-existent. This was typical in large assessment projects (assessments 4, 5, 7, Table S1-1). On the other hand, most researchers were happy in their own niche and did not expect that other experts could or should learn the details of their work. Consequently, the perceived need for shared tools or open data was often low, which hindered mutual sharing, learning and reuse.

The implementation phase of Climate Watch, which started in December 2018, also involved citizens, decision-makers and other municipalities. It was the largest case study so far using open policy practice. It combined existing and produced new climate emission models for municipalities. A long-term objective was to collect detailed input data ideally about the whole country and offer all models to all municipalities, thus maximising reuse.

An important skill in open policy practice was to learn to identify important pieces of relevant information (such as scientific facts, publications, discussions, etc.) and to add that information into a proper place in an insight network by using open policy ontology and a reasonable amount of work. The more there was user need for a piece of information, the more it was worth producing it. An ontology helped to do this in practice so that the output was understandable for both humans and computers.

The accumulation of scientific merit was a key motivator for researchers. Policy support work typically did not result in scientific articles. When researchers evaluated the efficiency of their own work, they preferred tasks that produced articles in addition to societal benefit. The same reasoning was seen with open assessments and knowledge crystals, resulting in a reluctance to participate. Win–win situations could be found if policy processes were actively developed into containing research aspects, so that new information would be produced for decision-makers but also for scientific audiences.

### Categories of interaction

Assessment methods have changed remarkably in 40 years. During the last decades, the trend has been from isolated to more open approaches but all categories of interaction (Table 6) are still in use [7]. The trend among the open assessments (Additional file 1: Appendix S1) seemed also to skew towards more participatory processes. Enabling participation was not enough, as interaction required facilitation and active invitation of decision-makers, experts, and stakeholders. Although openness and participation were available in all the open assessments in theory, only a minority of them actually had enough resources for facilitation to realise good co-creation in practice. In the first open assessments in the early 2000s, people were not familiar even with the concepts of co-creation. In recent examples, especially in the Helsinki climate strategy (assessment 8, Table S1-1), co-creation and openness were insisted on by decision-makers, civil servants and experts alike. There was also political will to give resources for co-creation and facilitation; this resulted in actual shared interaction between all groups.

The example in Helsinki produced interest and enthusiasm in both climate activists and other municipalities. The activists started to self-organise evaluation and monitoring using Climate Watch and ask explanations from civil servants whose actions were delayed. Several municipalities expressed their interest to start using Climate Watch in their own climate work, thus indicating that they had adopted the principles of openness and collaboration. This implies that, although the popularity of co-creation increased slowly during previous years, good experiences and awareness increase the rate of change, thus resulting in supra-linear progress in interaction.

### Test of shared understanding

Shared understanding clarified complex issues and elicited implicit valuations and reasonings in the open assessments. It facilitated rational discussion about a decision and explicated values of stakeholders, e.g. about vaccines (assessments 1, 2 in Table S1-1). It also created political pressure against options that were not well substantiated, e.g. about health effects of food (assessment 31, Table S1-1). Shared understanding was approached even when a stakeholder was ignorant of or even hostile to new insights, or not interested in participating, such as in trip aggregation assessment or health benefit–risk assessment of Baltic fish (assessments 11 and 16, Table S1-1). Then, there was an attempt to describe stakeholders’ views based on what other people know about their values. Everyone’s views are seen as important policy-relevant information that may inform decision-making.

Shared understanding was a well-accepted idea among many decision-makers in Finland. This was observed in collaboration with the Prime Minister’s Office of Finland (assessment 27, Table S1-1). Many civil servants in ministries liked the idea that sometimes it is better to aim to understanding rather than consensus. They soon adopted the easy version of the term and started to use it in their own discussions and publications [45, 46].

However, shared understanding was not unanimously accepted. Experts were often reluctant to start scientific discussions with citizens, especially if there were common or strong false beliefs about the topic among the public. In such cases, a typical argument was that the role of an expert is to inform and, if possible, suppress false beliefs rather than engage in producing common descriptions about differing views. The target seemed to be to convince the opponent rather than increase understanding among the audience.

The test of shared understanding was a useful tool to recognise when not all values, causal chains or decision-makers’ rationale were known and documented. Yet, a lack of time or resources often prevented further facilitation, information collection or expansion of the scope of an assessment.

## Discussion

This article presents methods and tools designed for decision support. Many of them have already been successfully used, while others have been identified as important parts of open policy practice but have not been extensively tested.

The discussion is organised around the five principles of open policy practice, namely collaboration, openness, causality, criticism and intentionality. The principles are looked at in the light of popularity, acceptance and lessons learned from practical experience.

The five principles are not unique for open policy practice; on the contrary, they have been borrowed from various disciplines (for reviews, see [1, 7]). The aim was to use solid principles to build a coherent set of methods that gives practical guidance to decision support. It is reassuring that many principles from the original collection [19] have increased in popularity in the society. There are also studies comparing parts of open policy practice to other existing methods [47].

The results showed that the methods connected the five principles quite well to the properties of good policy support (Table 8). Open collaboration indeed resulted in high-quality content when knowledge crystals, web-workspaces and co-creation were utilised. End-user interaction and structured discussions helped to revise scope and content, thus improving relevance and usability. Acknowledging disagreements and producing shared understanding created acceptability, and openly shared information objects, such as data and models, improved availability and efficiency.

The experiences about open policy practice demonstrate that it works as expected when the participants are committed to collaborating using the methods, practices and tools. However, there have been less participants in most open assessments than what had been hoped for. This can partly be affected by their own actions, as reader and contributor numbers clearly went up with active facilitation or marketing with large media coverage and public interest. Some other reasons cannot be easily affected directly, such as inertia to change established practices or lack of scientific merit. Thus, a major long-term challenge is to build an attractive assessor community, culture and incentives for decision support.

The GovLab in New York is an example of such activity (www.thegovlab.org). They have expert networks, training, projects and data sources available to improve policy support. There is a need for similar tools and training designed to facilitate a change elsewhere. New practices could also be promoted by developing ways to give scientific — or political — merit and recognition more directly based on online co-creation contributions. The current publication counts and impact factors — or public votes — are very indirect measures of scientific or societal importance of the information or policies produced.

Knowledge crystals offer a collaboration forum for updating scientific understanding about a topic in a quicker and easier way than publishing scientific articles. Knowledge crystals are designed to be updated based on continuous discussion about the scientific issues (or valuations, depending on the topic) aiming to back up conclusions. In contrast, scientific articles are expected to stay permanently unchanged after publication. Articles offer little room for deliberation about the interpretation or meaning of the results after a manuscript is submitted – reviewer comments are often not published, and further discussion about an article is rare and mainly occurs only if serious problems are found. Indeed, the current scientific publishing system is poor in correcting errors via deliberation [48].

Shared understanding is difficult to achieve if the decision-maker, media environment or some political groups are indifferent about or even hostile against scientific knowledge or public values. For many interest groups, non-public lobbying, demonstrations and even spreading faulty information are attractive ways of influencing the outcome of a decision. These are problematic methods from the perspective of open policy practice, because they reduce the availability of important information in decision processes.

Further studies are needed on how open, information-based processes could be developed to be more tempting to groups that previously have preferred other methods. A key question is whether shared understanding is able to offer acceptable solutions to disagreeing parties and alleviate political conflict. Another question is whether currently under-represented groups have better visibility in such open processes. Additionally, more information is needed about how hostile contributions get handled when they occur; fortunately, they were very rare in the open assessments.

There is no data about open policy practice usage in a hostile environment. Yet, open policy practice can be collaboratively used even without support from a decision-maker or an important stakeholder. Although their objective values are important for an assessment, these may be either deduced indirectly from their actions, or even directly replaced by the objectives of the society at large. Thus, open policy practice is arguably a robust set of methods that can be used to bypass non-democratic power structures and focus on the needs of the public even in a non-optimal collaboration environment.

There is still a lot to learn about using co-created information in decision-making. Experiences so far have demonstrated that decision-making can be more evidence-informed than what it typically is, and several tools promoting this change are available.

Openness in science is a guiding principle and a current megatrend, and its importance has been accepted much more widely during recent years. Yet, the practices in research are changing slowly, and many current practices are actually in conflict with openness. For example, it is common to hide expert work until it has been finalised and published, to publish in journals where content is not freely available, and to not open the data used.

A demand to produce assessments openly and describe all reasoning and data already from the beginning was often seen as an unreasonable requirement and made experts reluctant to participate. This observation raised two opposite conclusions – either that openness should be incentivised and promoted actively in all research and expert work [9], including decision support, or that openness as an objective hinders expert work and should be rejected. The latter conclusion was strong among experts in the early open assessments, but the former one has gained popularity.

There are several initiatives to open scientific processes, such as Open Science Foundation (www.osf.io). These are likely to promote change in science at large and indirectly also in scientific support of decision-making.

Among experts, causality was seen as a backbone of impact modelling. In political arenas, causal discourse was not as prominent, as it was often noticed that there was actually little solid information about the most policy-relevant causal chains, and therefore values dominated policy discussions. Climate Watch was the most ambitious endeavour in the study material to quantify all major causal connections of a climate action plan. The approach was supported by the city administration and stakeholders alike. Causal quantification created an additional resource need that was not originally budgeted. It is not yet known how Helsinki, other cities and research institutes will distribute the resources and tasks of causal modelling and information produced. Yet, actions in the national energy and climate plans total 260 billion euro per year in the EU [49]. Therefore, even minor improvements in the efficiency or effectiveness of climate actions would make causal assessments worthwhile.

Criticism has a central role in the scientific method. It is applied in practical situations because rejecting poor statements is easier and more efficient than trying to prove statements true [4]. Most critique in open assessments was verbal or as written discussion between participants, focussing on particular, often detailed topics. Useful information structures have been found for criticism, notably structured discussions that can target any part of an assessment (scope, data, premises, analyses, structure, results, etc.).

The current practices of open criticism in research are far from optimal, as criticism rarely happens. Pre-publishing peer review is almost the only time when scientific work is criticised by people outside the research group, and those are typically not open. A minute fraction of published works are criticised openly in journals; a poor work is simply not cited and subsequently forgotten. Interestingly, some administrative processes follow scientific principles better than many research processes do; for example, environmental impact assessment has a compulsory process for open criticism at both the design and result phases [50].

Intentionality requires that the objectives and values of stakeholders in general and decision-makers in particular are understood. In the studied assessments, some values were always identified and documented. However, it was not common to systematically describe all relevant values or even ensure that the assessed objectives were actually the most important ones for the decision-maker. There is clearly a need to prioritise facilitation and interaction about values.

In shared understanding, some claims were found unsubstantiated or clearly false. On the societal level, open policy practice aimed to increase political pressure against decisions based on poor ideas by explicating the problems and informing the public about them. The purpose was not to pressure individuals to reject their unsubstantiated thoughts. Personal beliefs were understood rather than threatened, because the aim was to build acceptance and facilitate contributions. However, it is not known what happens with very sensitive personal topics because there were no such issues in the studied assessments.

Politics in western democracies is typically based on a premise that, ultimately, the citizens decide about things by voting. Therefore, in a sense, people cannot vote ‘wrong’. In contrast, open policy practice is based on a premise that the objectives of the citizens are the ultimate guiding principle, and it is a matter of discussion, assessment and other information work to suggest which paths should or should not be taken to reach these objectives. This thinking is close to James Madison’s ideas about democracy in Federalist No. 63 from 1788 [51]. In this context, people vote wrong if they vote for an option that is incapable of delivering the outcomes that they want.

If people are well-informed and have the time and capability of considering different alternatives, the two premises lead to similar outcomes. However, recent policy research has shown that this prerequisite is often not met, and people can be and increasingly are being misled, especially with modern microtargeting tools [44]. The need for protecting people and decision-making from misleading information has been recognised.

Public institutions, such as the independent justice system, free press and honest civil servants, provide protection against misleading activities and disruptive policies. These democratic institutions have deteriorated globally and, in some countries particularly, even in places with a good track record [52].

Destructive policies may be an effective way to inform stakeholders in a grim societal environment. Open policy practice may not be very effective in choosing the best alternative among good ones, but it is probably more effective in identifying and rejecting poor alternatives, i.e. destructive policies, which is often more important. This is expected to result in more stable and predictable policies. It is possible to focus on disseminating information about what actions especially should not be taken, why and how it is known. In such discourse, the message can be practical, short, clear and rationale is available for anyone interested. Practical experiments are needed to tell whether this could reduce the support of destructive policies among the public.

Further research is also needed to study other aspects of destructive policies – can such policies be unambiguously recognised? Is shared understanding about them convincing enough among decision-makers to change policies? Does it cause objections about science being biased and partisan? Does open policy practice prevent destructive policies from gaining political support?

## Conclusions

In conclusion, open policy practice works technically as expected. Open assessments can be performed openly online. They do not fail due to the reasons many people think they will, namely low quality contributions, malevolent attacks or chaos caused by too many uninformed participants; these phenomena are very rare. Shared understanding has proved to be a useful concept that guides policy processes toward a more collaborative approach, whose purpose is wider understanding rather than winning.

However, open policy practice has not been adopted in expert work or decision support as expected. A key hindrance has been that the initial cost of learning and adopting new tools and practices has been higher than what an expert is willing to pay for participation in a single assessment, even if its impacts on the overall process are positive. The increased availability, acceptability and inter-assessment efficiency have not yet been fully recognised by the scientific or policy community.

Active facilitation, community building and improving the user-friendliness of the tools were identified as key solutions in improving the usability of the method in the future.

## Supplementary information

**Additional file 1: Appendix S1.** Open assessments performed. **Appendix S2.** Examples of insight networks. **Appendix S3.** Open policy ontology. **Appendix S4.** Workspace tools: OpasnetUtils package and Opasnet Base. **Appendix S5.** Tools to help in shared understanding.

## Data Availability

The datasets generated and/or analysed during the current study are available at the Opasnet repository, http://en.opasnet.org/w/Open_policy_practice.

## Footnotes

1 This paper has its foundations on environmental health, but the idea of decision support necessarily looks at aspects seen relevant from the point of view of the decision-maker, not from that of an expert in a particular field. Therefore, this article as well as the method described are deliberately taking a wide view, covering all areas of expertise. However, all practical case studies have their main expertise needs in public health, and often specifically in environmental health.

2 Whenever this article presents a term in italic (e.g. *open assessment*), it indicates that there is a page at the Opasnet web-workspace describing that term and that it can be accessed using a respective link (e.g. http://en.opasnet.org/w/Open_assessment).

3 Insight network was originally called *pyrkilo* (and at some point also ‘extended causal diagram’). The word and concept *pyrkilo* were coined in 1997. In Finnish, *pyrkilö* means ‘an object or process that tends to produce or aims at producing certain kinds of products’. The reasoning for using the word was that *pyrkilo* diagrams and related structured information, such as models, tend to improve understanding and thus decisions. The first wiki website was also called Pyrkilo, but the name was soon changed to Opasnet.
