# Supplementary material for: From insight network to open policy practice: practical experiences
Source: Health Res Policy Syst. 2020 Apr 3;18:36. doi: 10.1186/s12961-020-00547-3 (PMC7118856; doi:10.1186/s12961-020-00547-3)
Supplement: Supplementary file 1 — Additional file 1: Appendix S1. Open assessments performed. Appendix S2. Examples of insight networks. Appendix S3. Open policy ontology. Appendix S4. Workspace tools: OpasnetUtils package and Opasnet Base. Appendix S5. Tools to help in shared understanding. [file 12961_2020_547_MOESM1_ESM.pdf]

From insight network to open policy practice: practical experiences

Jouni T. Tuomisto, Mikko Pohjola, Teemu Rintala

Appendix S1: Open assessments performed

A number of open assessments have been performed in several research projects (see the funding declaration) and health assessments since 2004. Some assessments have also been done on international *Kuopio Risk Assessment Workshops* for doctoral students in 2007, 2008, and 2009 and on a Master's course *Decision Analysis and Risk Management* (6 credit points), organised by the University of Eastern Finland (previously University of Kuopio) in 2011, 2013, 2015, and 2017.

More assessments can be found at Opasnet page *Category:Assessments*.

Table S1-1. Some environmental health assessments performed using open assessment. References give links to both an assessment page and a scientific publication as applicable.

Topic	#	Assessment	Year	Project
Vaccine effectiveness and safety	1	Assessment of the health impacts of H1N1 vaccination[1]	2011	In-house, collaboration with Decision Analysis and Risk Management course
	2	Tendering process for pneumococcal conjugate vaccine[2]	2014	In-house, collaboration with the National Vaccination Expert Group
Energy production, air pollution and climate change	3	Helsinki energy decision[3]	2015	In-house, collaboration with city of Helsinki
	4	Climate change policies and health in Kuopio[4]	2014	Urgenche, collaboration with city of Kuopio
	5	Climate change policies in Basel[5]	2015	Urgenche, collaboration with city of Basel
	6	Availability of raw material for biodiesel production[6]	2012	Jatropha, collaboration with Neste Oil
	7	Health impacts of small scale wood burning[7]	2011	Bioher, Claih
	8	Climate strategy of Helsinki: Carbon neutral Helsinki 2035 action plan[8]	2018	In-house, collaboration with city of Helsinki
	9	Climate mitigation of the social affairs and health sector in Finland[9]	2020	In-house, commissioned by the Prime Minister
Health, climate, and	10	Gasbus - health impacts of Helsinki bus traffic[10]	2004	Collaboration with Helsinki Metropolitan Area

Topic	#	Assessment	Year	Project
economic effects of traffic	11	Cost-benefit assessment on composite traffic in Helsinki[11]	2005	In-house
Risks and benefits of fish consumption	12	Benefit-risk assessment of Baltic herring in Finland[12]	2015	Collaboration with Finnish Food Safety Authority
	13	Benefit-risk assessment of methyl mercury and omega-3 fatty acids in fish[13]	2009	Beneris
	14	Benefit-risk assessment of fish consumption for Beneris[14]	2008	Beneris
	15	Benefit-risk assessment on farmed salmon[15]	2004	In-house
	16	Benefit-risk assessment of Baltic herring and salmon intake[16]	2018	BONUS GOHERR
Dioxins, fine particles	17	TCDD: A challenge to mechanistic toxicology[17]	1999	EC ENV4-CT96-0336
	18	Comparative risk assessment of dioxin and fine particles[18]	2007	Beneris
Plant-based food supplements	19	Compound intake estimator[19]	2014	Plantlibra
Health and ecological risks of mining	20	Paakkila asbestos mine[20]	1999	In-house
	21	Model for site-specific health and ecological assessments in mines[21]	2013	Minera
	22	Risks of water from mine areas [22]	2018	Kaveri
Water safety	23	Water Guide for assessing health risks of drinking water contamination[23]	2013	Conpat
	24	Bathing Water Guide for assessing health risks of bathing water contamination[24]	2019	Water Guide update
Organisational assessments	25	Analysis and discussion about research strategies or organisational changes within THL	2017	In-house
	26	Transport and communication strategy in digital Finland[25]	2014	Collaboration with the Ministry of Transport and Communications of Finland

Topic	#	Assessment	Year	Project
Information use in government or municipality decision support	27	Case studies: Assessment of immigrants' added value; Real-time co-editing, Fact-checking, Information design[26]	2016	Yhtäköyttä, collaboration with Prime Minister's Office
	28	Evaluation of forest strategy process for Puijo, Kuopio[27]	2012	In-house
Indicator development	29	Environmental health indicators in Finland[28]	2018	Ympäristöterveysindikaattori
Structuring discussions	30	Developing and testing tools and practices for structured argumentation[29]	2019	Citizen Crystal
Food safety and diet	31	Health risks of chemical and microbial contaminants and dietary factors in food in Finland[30]	2019	Ruori, collaboration with e.g. Ministry of Agriculture and Prime Minister's Office

References for assessments

1. Assessment: http://en.opasnet.org/w/Assessment_of_the_health_impacts_of_H1N1_vaccination. Accessed 1 Feb 2020.
2. Assessment: http://en.opasnet.org/w/Tendering_process_for_pneumococcal_conjugate_vaccine. Accessed 1 Feb 2020.
3. Tuomisto JT, Rintala J, Ordén P, Tuomisto HM, Rintala T. Helsingin energiapäätös 2015. Avoin arviointi terveys-, ilmasto- ja muista vaikutuksista. [Helsinki energy decision 2015. An open assessment on health, climate, and other impacts]. Helsinki: National Institute for Health and Welfare. Discussionpaper 24; 2015. http://urn.fi/URN:ISBN:978-952-302-544-8 Assessment: http://en.opasnet.org/w/Helsinki_energy_decision_2015. Accessed 1 Feb 2020.
4. Asikainen A, Pärjälä E, Jantunen M, Tuomisto JT, Sabel CE. Effects of Local Greenhouse Gas Abatement Strategies on Air Pollutant Emissions and on Health in Kuopio, Finland. Climate 2017;5(2):43; doi:10.3390/cli5020043 Assessment: http://en.opasnet.org/w/Climate_change_policies_and_health_in_Kuopio. Accessed 1 Feb 2020.
5. Tuomisto JT, Niittynen M, Pärjälä E, Asikainen A, Perez L, Trüeb S, Jantunen M, Künzli N, Sabel CE. Building-related health impacts in European and Chinese cities: a scalable assessment method. Environmental Health 2015;14:93. doi:10.1186/s12940-015-0082-z Assessment: http://en.opasnet.org/w/Climate_change_policies_in_Basel. Accessed 1 Feb 2020.
6. Sandström V, Tuomisto JT, Majaniemi S, Rintala T, Pohjola MV. Evaluating effectiveness of open assessments on alternative biofuel sources. Sustainability: Science, Practice & Policy 2014;10;1. doi:10.1080/15487733.2014.11908132 Assessment: http://en.opasnet.org/w/Biofuel_assessments. Accessed 1 Feb 2020.
7. Taimisto P, Tainio M, Karvosenoja N, Kupiainen K, Porvari P, Karppinen A, Kangas L, Kukkonen J, Tuomisto JT. Evaluation of intake fractions for different subpopulations due to

primary fine particulate matter (PM2.5) emitted from domestic wood combustion and traffic in Finland. Air Quality Atmosphere and Health 2011;4:3-4:199-209. doi:10.1007/s11869-011-0138-3 Assessment: http://en.opasnet.org/w/BIOHER_assessment. Accessed 1 Feb 2020.

8. City of Helsinki. The Carbon-neutral Helsinki 2035 Action Plan. Publications of the Central Administration of the City of Helsinki 2018:4. http://carbonneutralcities.org/wp-content/uploads/2019/06/Carbon_neutral_Helsinki_Action_Plan_1503019_EN.pdf Assessment: https://ilmastovahti.hel.fi. Accessed 1 Feb 2020.
9. Tuomisto JT. (2020) Climate emissions of the social affairs and health sector in Finland and potential mitigation actions. Assessment: https://hnpolut.dokku.teamy.fi. Accessed 1 Feb 2020
10. Tainio M, Tuomisto JT, Hanninen O, Aarnio P, Koistinen, KJ, Jantunen MJ, Pekkanen J. Health effects caused by primary fine particulate matter (PM2.5) emitted from buses in the Helsinki metropolitan area, Finland. RISK ANALYSIS 2005;25:1:151-160. Assessment: http://en.opasnet.org/w/Gasbus_-_health_impacts_of_Helsinki_bus_traffic. Accessed 1 Feb 2020.
11. Tuomisto JT; Tainio M. An economic way of reducing health, environmental, and other pressures of urban traffic: a decision analysis on trip aggregation. BMC PUBLIC HEALTH 2005;5:123. http://biomedcentral.com/1471-2458/5/123/abstract Assessment: http://en.opasnet.org/w/Cost-benefit_assessment_on_composite_traffic_in_Helsinki. Accessed 1 Feb 2020.
12. Tuomisto JT, Niittynen M, Turunen A, Ung-Lanki S, Kiviranta H, Harjunpää H, Vuorinen PJ, Rokka M, Ritvanen T, Hallikainen A. Itämeren silakka ravintona – Hyöty-haitta-analyysi. [Baltic herring as food - a benefit-risk analysis] ISBN 978-952-225-141-1. Helsinki: Eviran tutkimuksia 1; 2015 (in Finnish). Assessment: http://fi.opasnet.org/fi/Silakan_hy%C3%B6ty-riskiarvio. Accessed 1 Feb 2020.
13. Leino O, Karjalainen AK, Tuomisto JT. Effects of docosahexaenoic acid and methylmercury on child's brain development due to consumption of fish by Finnish mother during pregnancy: A probabilistic modeling approach. Food Chem Toxicol. 2013;54:50-8. doi:10.1016/j.fct.2011.06.052. Assessment: http://en.opasnet.org/w/Benefit-risk_assessment_of_methyl_mercury_and_omega-3_fatty_acids_in_fish. Accessed 1 Feb 2020.
14. Gradowska PL. Food Benefit-Risk Assessment with Bayesian Belief Networks and Multivariable Exposure-Response. Delft: Delft University of Technology (doctoral dissertation); 2013. https://repository.tudelft.nl/islandora/object/uuid:9ced4cb2-9809-4b58-af25-34e458e8ea23/datastream/OBJ Assessment: http://en.opasnet.org/w/Benefit-risk_assessment_of_fish_consumption_for_Beneris. Accessed 1 Feb 2020.
15. Tuomisto JT, Tuomisto J, Tainio M, Niittynen M, Verkasalo P, Vartiainen T et al. Risk-benefit analysis of eating farmed salmon. Science 2004;305(5683):476. Assessment: http://en.opasnet.org/w/Benefit-risk_assessment_on_farmed_salmon. Accessed 1 Feb 2020.
16. Tuomisto JT, Asikainen A, Meriläinen P et Haapasaari P. Health effects of nutrients and environmental pollutants in Baltic herring and salmon: a quantitative benefit-risk assessment. BMC Public Health 20, 64 (2020). https://doi.org/10.1186/s12889-019-8094-1 Assessment:

http://en.opasnet.org/w/Goherr_assessment, data archive: https://osf.io/brxpt/. Accessed 1 Feb 2020.
17. Tuomisto JT. TCDD: a challenge to mechanistic toxicology [Dissertation]. Kuopio: National Public Health Institute A7; 1999.
18. Leino O, Tainio M, Tuomisto JT. Comparative risk analysis of dioxins in fish and fine particles from heavy-duty vehicles. Risk Anal. 2008;28(1):127-40. Assessment: http://en.opasnet.org/w/Comparative_risk_assessment_of_dioxin_and_fine_particles. Accessed 1 Feb 2020.
19. Assessment: http://en.opasnet.org/w/Compound_intake_estimator. Accessed 1 Feb 2020.
20. Tuomisto JT, Pekkanen J, Alm S, Kurttio P, Venäläinen R, Juuti S et al. Deliberation process by an explicit factor-effect-value network (Pyrkilo): Paakkila asbestos mine case, Finland. Epidemiol 1999;10(4):S114.
21. Kauppila T, Komulainen H, Makkonen S, Tuomisto JT, editors. Metallikaivosalueiden ympäristöriskinarviointiosaamisen kehittäminen: MINERA-hankkeen loppuraportti. [Summary: Improving Environmental Risk Assessments for Metal Mines: Final Report of the MINERA Project.] Helsinki: Geology Survey Finland, Research Report 199; 2013. 223 p. ISBN 978-952-217-231-0. Assessment: http://fi.opasnet.org/fi/Minera-malli. Accessed 1 Feb 2020.
22. Assessment: http://fi.opasnet.org/fi/Kaivosvesien_riskit_(KAVERI-malli). Accessed 1 Feb 2020.
23. Assessment: http://en.opasnet.org/w/Water_guide. Accessed 1 Feb 2020.
24. Assessment: http://en.opasnet.org/w/Bathing_water_guide. Accessed 1 Feb 2020.
25. Liikenne ja viestintä digitaalisessa Suomessa. Liikenne- ja viestintäministeriön tulevaisuuskatsaus 2014 [Transport and and communication in digital Finland] Helsinki: Ministry of Transport and Communication; 2014. http://urn.fi/URN:ISBN:978-952-243-420-3 Assessment: http://fi.opasnet.org/fi/Liikenne_ja_viestint%C3%A4_digitaalisessa_Suomessa_2020. Accessed 1 Feb 2020.
26. Tuomisto JT, Muurinen R, Paavola J-M, Asikainen A, Ropponen T, Nissilä J. Tiedon sitominen päätöksentekoon. [Binding knowledge to decision making] Helsinki: Publications of the Government's analysis, assessment and research activities 39; 2017. ISBN 978-952-287-386-6 http://tietokayttoon.fi/julkaisu?pubid=19001. Assessment: http://fi.opasnet.org/fi/Maahanmuuttoarviointi. Accessed 1 Feb 2020.
27. Kajanus M, Ollikainen T, Partanen J, Vänskä I. Kävijätutkimukseen perustuva Puijon virkistysmetsien hoito- ja käyttösuunnitelma. [Forest strategy for recreational forests at Puijo, Kuopio, based on visitor study.] (in Finnish) Kuopion kaupunki, Metsätoimisto; 2010. http://fi.opasnet.org/fi-opwiki/images/8/8a/Puijo-loppuraportti.pdf. Assessment: http://fi.opasnet.org/fi/Puijon_metsien_k%C3%A4ytt%C3%B6suunnitelman_p%C3%A4%C3%A4t%C3%B6ksenteko Accessed 1 Feb 2020.
28. Tuomisto JT, Asikainen A, Korhonen A, Lehtomäki H. Teemasivu ympäristöterveys [Portal: Environmental health]. A website, THL, 2018. [1]
29. Hastrup T. Knowledge crystal argumentation tree. https://dev.tietokide.fi/?Q10. Web tool. Accessed 1 Feb 2020.

30. Suomi J, Haario, P et al. Costs and Risk Assessment of the Health Effects of the Food System. Publications of the Government´s analysis, assessment and research activities 2019:64. http://urn.fi/URN:ISBN:978-952-287-797-0. Accessed 1 Feb 2020.

Appendix S2: Examples of insight networks

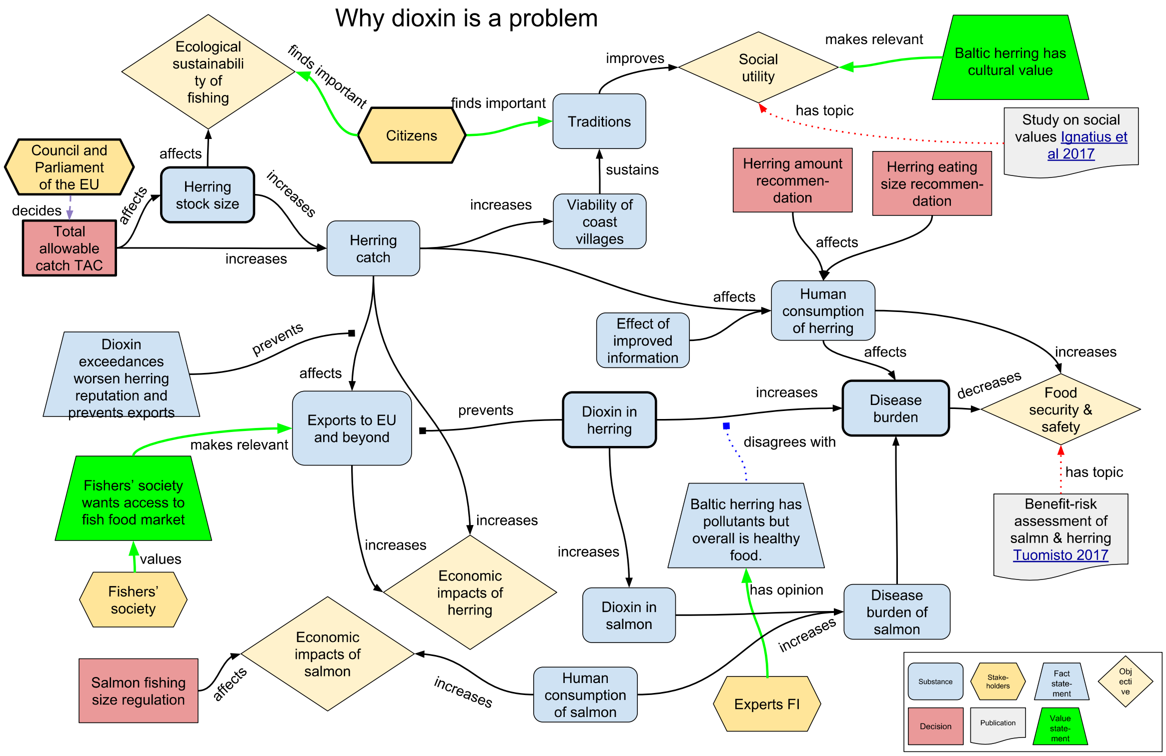

Figure S2-1. Insight network about dioxins in Baltic fish. The focus is on reasoning and value judgements and their connections to causal chains about dioxins and health.

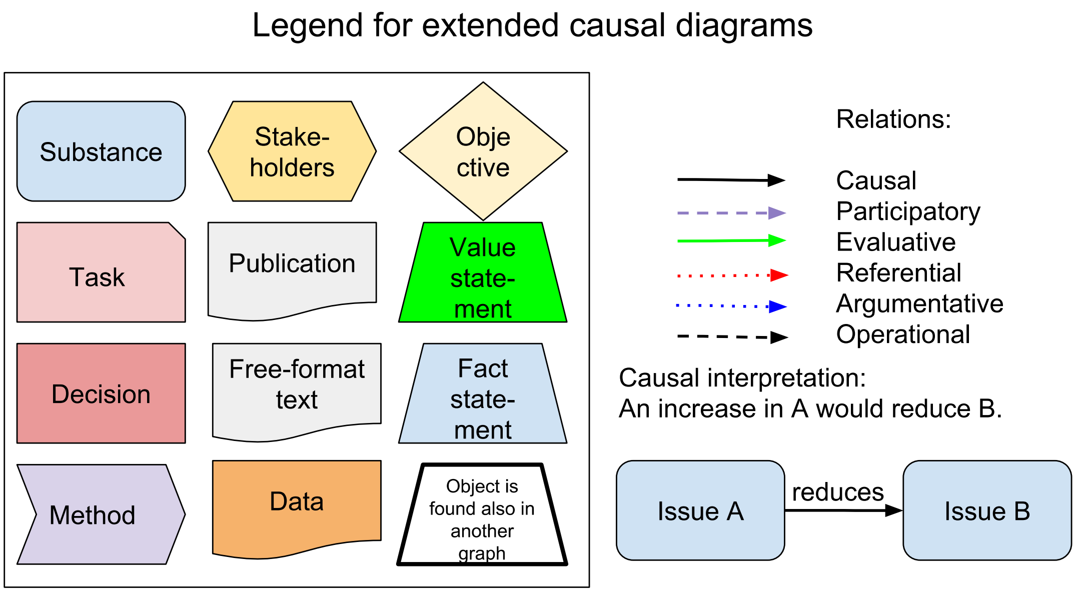

Figure S2-2. Legend for main object types used in insight networks. The actual colours and formatting depend on the capabilities of the software used.

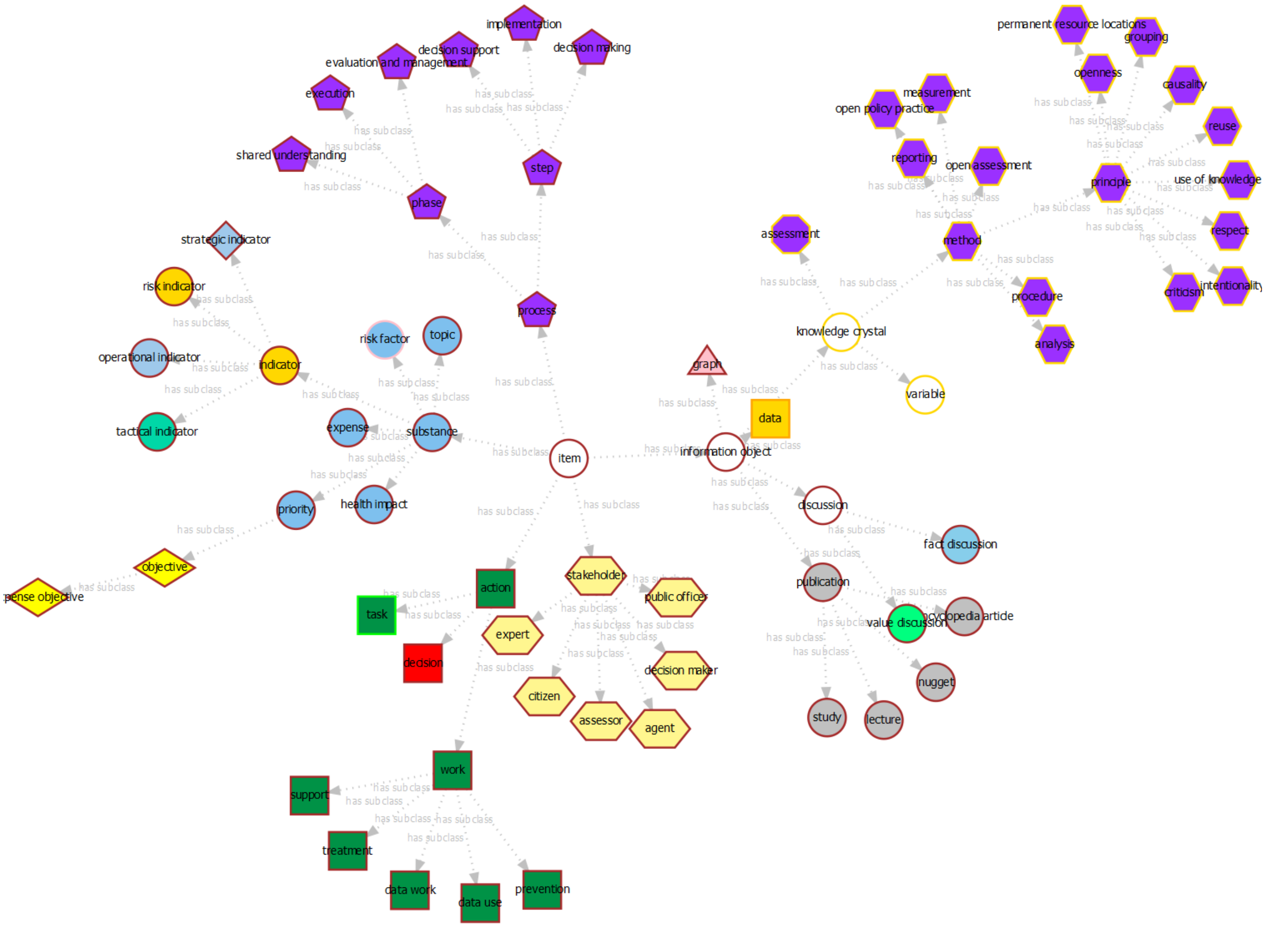

Figure S2-3. Items in open policy ontology shown in an insight network format. All relations are of types 'has subclass' or 'has part'.

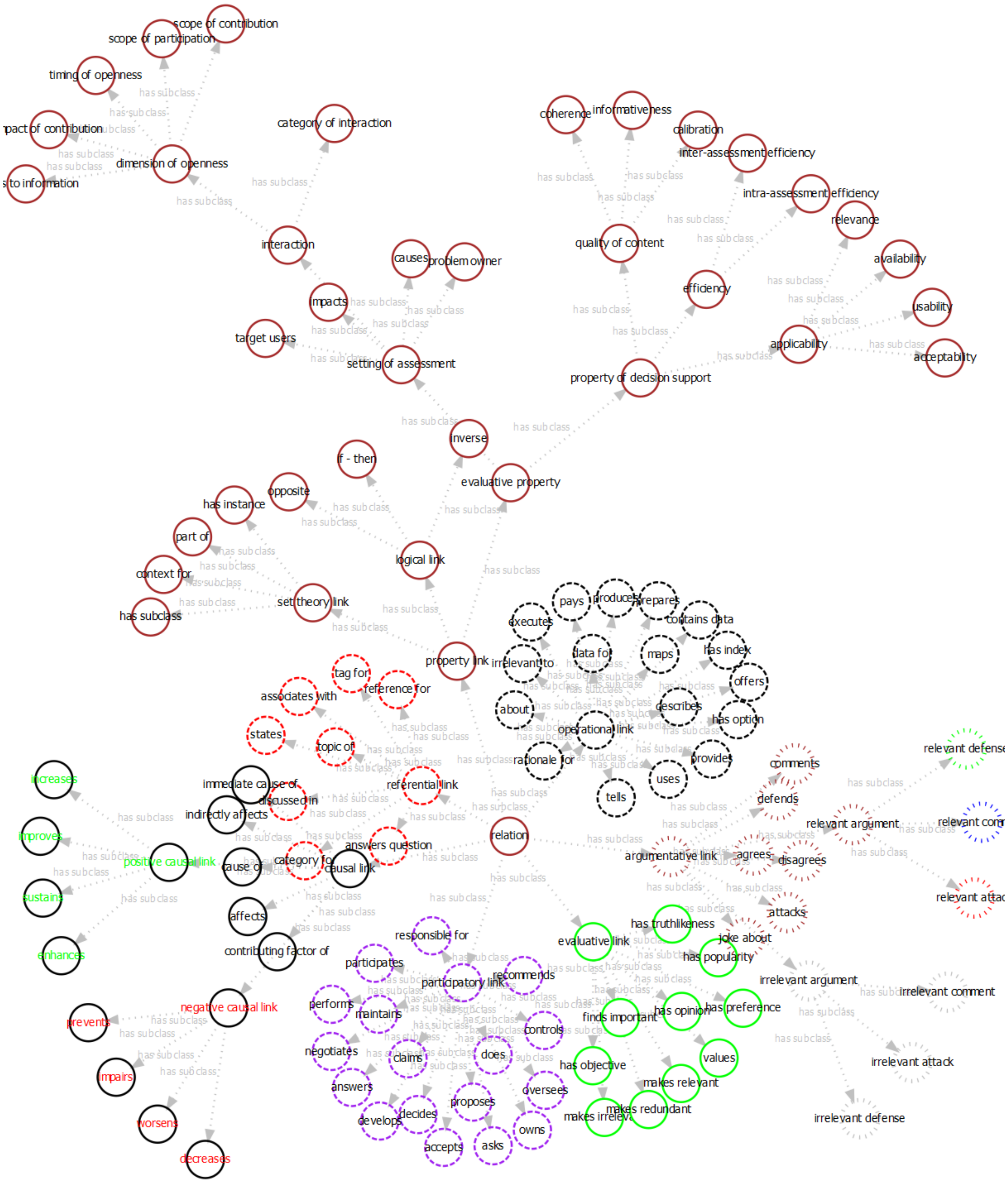

Figure S2-4. Relations in open policy ontology shown in an insight network format. In this graph, relations are shown as nodes, and the arcs between relations are of types 'has subclass', 'has part', or 'inverse'.

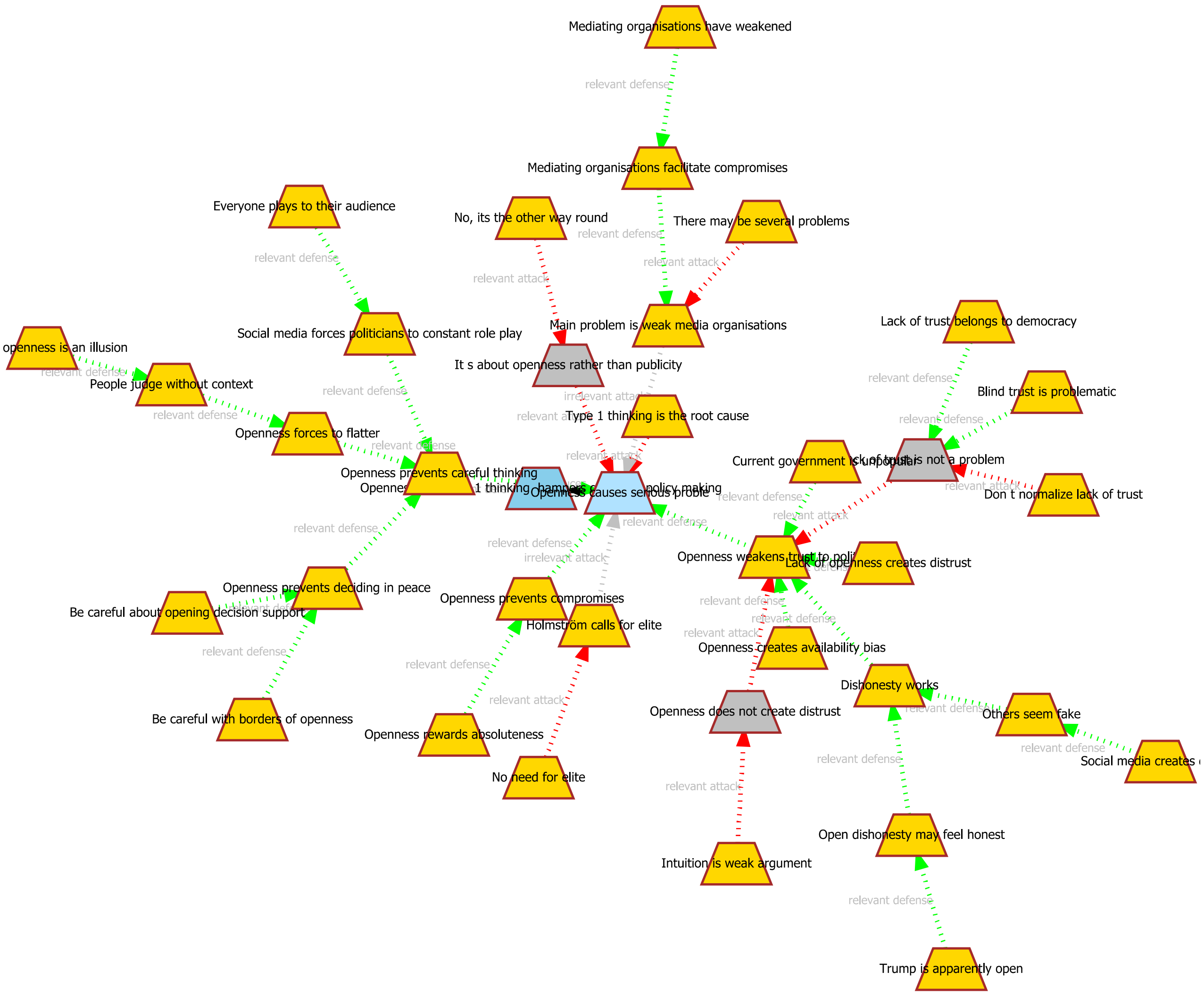

Figure S2-5. Structured discussion about risks of open governance. The original discussion was held in Finnish and can be found from http://fi.opasnet.org/fi/Keskustelu:Jaettu_ymm%C3%A4rrys. Trapezoids are statements. Light blue is an opening fact statement ('Openness causes serious problems to the quality of policy making.'), and blue is closing fact statement that is updated based on the discussion ('Openness causes serious problems to the quality of policy making, if it is too tightly connected to impulsive thinking in social media.'). Orange arguments are true and gray are false. Red arrow is an attack, green is a defense, and gray is irrelevant (Accessed 1 Feb 2020).

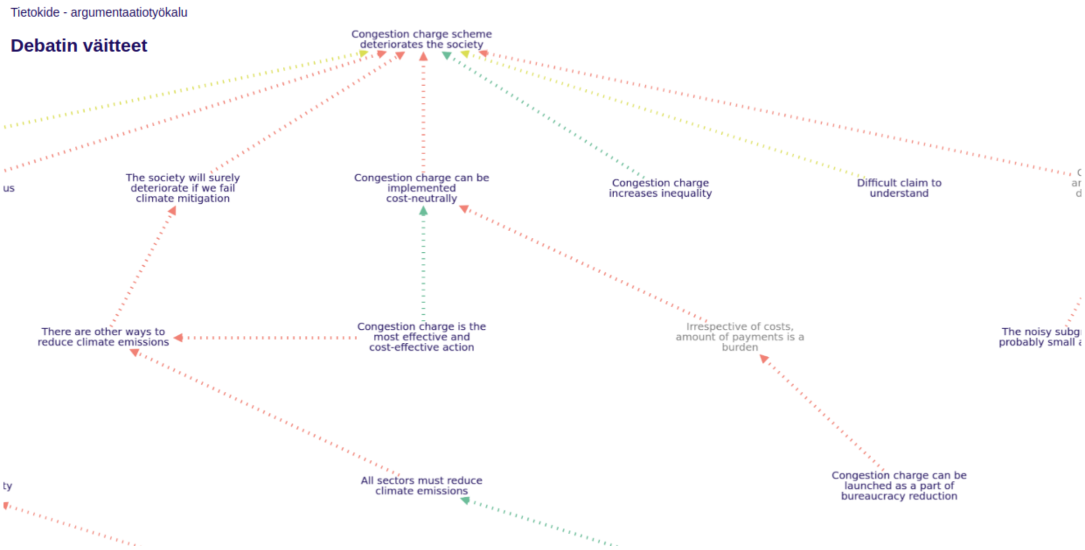

Figure S2-6. Structured discussion at an argumentation tool (https://dev.tietokide.fi/?Q10. Accessed 1 Feb 2020)

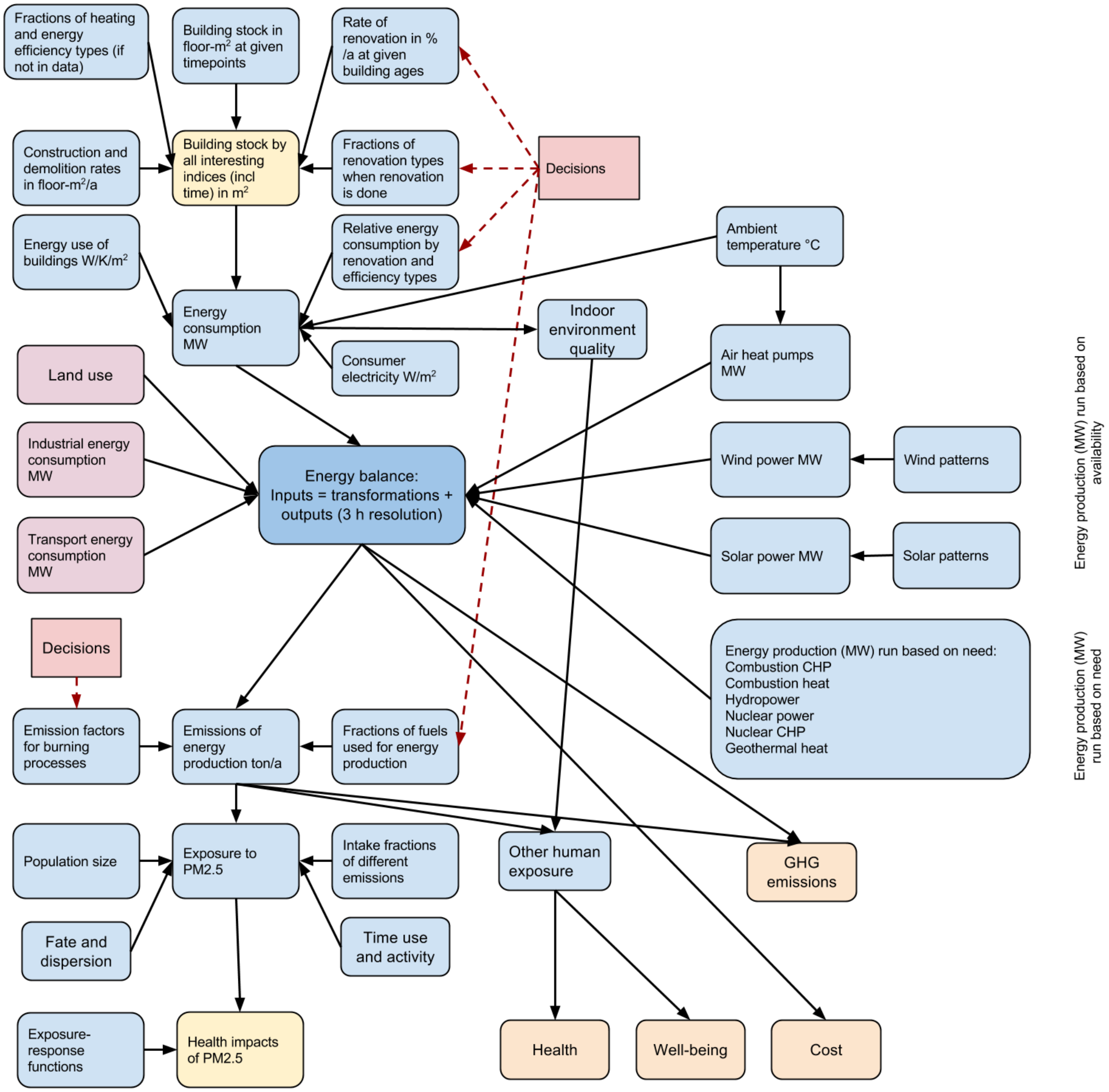

Figure S2-6. Insight network about the assessment model for Helsinki energy decision 2015.

Appendix S3: Open policy ontology

Shared understanding aims at producing a description of different views, opinions, and facts related to a specific topic such as a decision process. The open policy ontology describes the information structures that are needed to document shared understanding of a complex decision situation. The purpose of the structure is to help people identify hidden premises, beliefs, and values and explicate possible discrepancies. This is expected to produce better understanding among participants.

The basic structure of a shared understanding is a network of items and relations between them. This network uses Resource description framework, which is an ontology standard used to describe many Internet contents. Items and relations (aka properties) are collectively called resources. Each item is typically of one of the types mentioned below. This information is documented using property **instance of** (e.g. Goherr assessment is instance of assessment).

Items are written descriptions of the actual things (people, tasks, publications, or phenomena), and on this page these descriptions rather than the actual things are discussed. Different item types have different levels of standardisation and internal structure. For example, knowledge crystals are web pages that always have headings question, answer and rationale, and the information is organised under those headings. Some other items describe e.g. statements that are free-text descriptions about how a particular thing is or should be (according to a participant), and yet some others are metadata about publications. A common feature is that all items contain information that is relevant for a decision.

In the open policy ontology, each item may have lengthy texts, graphs, analyses or even models inside them. However, the focus here is on how the items are related to each other. The actual content is often referred to as one key sentence only (description). Each item also has a unique identifier URI that is used for automatic handling of data.

The most important items are knowledge crystals and they are described here.

- **Assessment** describes a particular decision situation and focuses on estimating impacts of different options. Its purpose is to support the making of that decision. Unlike other knowledge crystals, assessments typically have a defined start and end dates and they are closed after the decision is made. They also have contextually and situationally defined goals`to be able to better serve the needs of the decision makers of the decision.
- **Variable** answers a particular factual or ethical question that is typically needed in one or more assessments. The answer of a variable is continually updated as new information arises, but its question remains constant in time. Variable is the basic building block of assessments. In R, variables are typically implemented using ovariable objects from OpasnetUtils package.
- **Method** tells how to systematically implement a particular information task. Method is the basic building block for describing the assessment work (not reality, like variables). In practice, methods are "how-to-do" descriptions about how information should be produced, collected, analysed, or synthesised in an assessment. Typically, methods contain a software code or another algorithm to actually perform the method easily. In R, methods are typically ovariables

that require some context-specific upstream information about dependencies before it can be calculated.

There are also other important classes of items:

- **Publication** is any documentation that contains useful information related to a decision. Publications that are commonly used at Opasnet include encyclopedia article, lecture, nugget, and study. Other publications at Opasnet are typically uploaded as files.
  - **Encyclopedia article** is an object that describes a topic like in Wikipedia rather than answers a specific research question. They do not have a predefined attribute structure.
  - **Lecture**: Lecture contains a piece of information that is to be mediated to a defined audience and with a defined learning objective. It can also be description of a process during which the audience learns, instead of being a passive recipient of information.
  - **Nugget** is an object that is not editable by other people than a dedicated author (group) and is not expected to be updated once finalised. They do not have a predefined attribute structure.
  - **Study** describes a research study and its answers, i.e. observational or other data obtained in the study. The research questions are described as the question of the information object, and the study methods are described as the rationale of the object. Unlike in an article, introduction or discussion may be missing, and unlike in a variable, the answer and rationale of the study are more or less fixed after the work is done; this is because the interpretations of the results typically happen elsewhere, e.g. in variables for which a study contains useful information.
- **Discussion** is a hierarchically structured documentation of a discussion about a defined statement or statements.
- **Stakeholder** page is used to describe a person or group that is relevant for a decision or decision process; they may be an actor that has an active role in decision making or is a target of impacts. Contributors of Opasnet are described on their own user pages; other stakeholders may have their page on the main namespace.
- **Process** describes elements of a decision process.
- **Action** describes what, who and when should act to e.g. perform an assessment, make a decision, or implement policies.

Relations show different kinds of connections between items.

- **Causal link** tells that the subject may change the object (e.g. affects, increases, decreases, prevents).
- **Participatory link** describes a stakeholder's particular role related to the object (participates, negotiates, decides).
- **Operational link** tells that the subject has some kind of practical relation to the object (executes, offers, tells).
- **Evaluative link** tells that the subject shows preference or relevance about the object (has truthlikeness, value, popularity, finds important).

- **Referential link** tells that the object is used as a reference of a kind for the subject (makes relevant; associates to; has reference, has tag, has category).
- **Argumentative link** occurs between statements that defend or attack each other (attack, defend, comment).
- **Property link** connects an evaluative (acceptability, usability), a logical (opposite, inverse) or set theory (has subclass, has part) property to the subject.

Item types

This ontology is specifically about decision making, and therefore actions (and decisions to act) are handled explicitly. However, any natural, social, ethical or other phenomena may relate to a decision and therefore the vocabulary has to be very generic.

Table S3-1. Item types used in open policy ontology.

Class	English name	Finnish name	Description
	resource	resurssi	All items and relations are resources
resource	item	asia	Relevant pieces of information related policy making. Sometimes also refers to the real-life things that the information is about. Items are shown as nodes in insight networks.
resource	relation	relaatio	Information about how items are connected to each other. Relations are shown as edges in insight networks.
item	substance	ilmiö	Items about a substantive topic or phenomenon itself: What issues relate to a decision? What causal connections exist between issues? What scientific knowledge exist about the issues? What actions can be chosen? What are the impacts of these actions? What are the objectives and how can they be reached? What values and preferences exist?
item	stakeholder	sidosryhmä	Items about people or organisations who have a particular role in a policy process, either as actors or targets of impacts: Who participates in a policy process? Who should participate? Who has necessary skills for contributing? Who has the authority to decide? Who is affected by a decision?
item	process	prosessi	Items about doing or happening in relation with a topic, especially information about how a decision will be made): What will be decided? When will it be decided? How is the decision prepared? What political realities and restrictions exist?

Class	English name	Finnish name	Description
item	action	toiminta	Items about organising decision support (impact assessment, decision making, implementation, and evaluation): What tasks are needed to collect and organise necessary information? How is information work organised? How and when are decisions implemented? Actions are also important afterwards to distribute merit and evaluate the process: Who did what? How did information evolve and by whom?
item	information object	tieto-olio	A specified structure containing information about substance, stakeholders, processes, methods, or actions.
informatio n object	knowledge crystal	tietokide	information object with a standardised structure and contribution rules
knowledge crystal	assessment	arviointi	Describes a decision situation and typically provides relevant information to decision makers before the decision is made (or sometimes after the decision about its implementation or success). It is mostly about the knowledge work, i.e. tasks for decision support.
knowledge crystal	variable	muuttuja	Describes a real-world topic that is relevant for the decision situation. It is about the substance of the topic.
knowledge crystal	method	metodi	Describes how information should be managed or analysed so that it answers the policy-relevant questions asked. How to perform information work? What methods are available for a task? How to participate in a decision process? How to use statistical and other methods and tools? How to motivate participation? How to measure merit of contributions?
informatio n object	discussion part	keskustelun osa	Information object that is used to organise discussions into a specified structure. The purpose of the structure is to help validation of statements and facilitate machine learning.
informatio n object	discussion	keskustelu	Discussion, or structured argumentation, describes arguments about a particular statement and a synthesis about an acceptable statement. In a way, discussion is (a documentation of) a process of analysing the validity of a statement.
discussion	fact discussion	faktakes-kustelu	Discussion that can be resolved based on scientific knowledge.
discussion	value discussion	arvokes-kustelu	Discussion that can be resolved based on ethical knowledge.
discussion part	statement	väite	Proposition claiming that something is true or ethically good. A statement may be developed in a discussion by adding and organising related argumentation (according to pragma-dialectics), or by organising premises and inference rules (according to Perelman).

Class	English name	Finnish name	Description
statement	value statement	arvoväite	Proposition claiming that something is ethically good, better than something else, prioritised over something, or how things should be.
statement	fact statement	faktaväite	Proposition claiming how things are or that something is true.
value statement	true value statement	tosi arvoväite	A statement that has not been successfully invalidated.
value statement	false value statement	epätosi arvoväite	A statement that has been successfully invalidated.
fact statement	true fact statement	tosi faktaväite	
fact statement	false fact statement	epätosi faktaväite	
statement	true statement	tosi väite	
statement	false statement	epätosi väite	
statement	opening statement	avausväite	A statement that is the basis for a structured discussion, a priori statement.
statement	closing statement	lopetusväite	A statement that is the resolution of a structured discussion, a posteriori statement. Closing statement becomes an opening statement when the discussion is opened again.
opening statement	fact opening statement	avausfakta-väite	
closing statement	fact closing statement	lopetusfakta-väite	
opening statement	value opening statement	avausarvo-väite	
closing statement	value closing stetement	lopetusarvo-väite	
discussion part	argument	argumentti	A statement that has also contains a relation to its target as an integral part. Due to this relation, arguments appear inside discussions and target directly or indirectly the opening statement.
discussion part	argumentati on	väittely	Hierarchical list of arguments related to a particular statement.

Class	English name	Finnish name	Description
informatio n object	knowledge crystal part	tietokideosa	This is shown separately to illustrate that the objects are actually linked by has part rather than has subclass relation.
knowledge crystal part	question	kysymys	A research question asked in a knowledge crystal. The purpose of a knowledge crystal is to answer the question.
knowledge crystal part	answer	vastaus	An answer or set of answers to the question of a knowledge crystal, based on any relevant information and inference rules.
knowledge crystal part	rationale	perustelut	Any data, discussions, calculations or other information needed to convince a critical rational reader that the answer of a knowledge crystal is good.
knowledge crystal part	answer part	vastausosa	This is shown separately to illustrate that the objects are actually linked by has part rather than has subclass relation.
answer part	result	tulos	The actual, often numerical result to the question, conditional on relevant indices.
answer part	index	indeksi	A list of possible values for a descriptor. Typically used in describing the result of an ovariable.
answer part	conclusion	päätelmä	In an assessment, a textual interpretation of the result. Typically a conclusion is about what decision options should or should not be rejected and why based on the result.
knowledge crystal part	ovariable	ovariable	A practical implementation of a knowledge crystal in modelling code. Ovariable takes in relevant information about data and dependencies and calculates the result. Typically implemented in R using OpasnetUtils package and ovariable object type.
ovariable	key ovariable	avainova-riable	An ovariable that is shown on an insight network even if some parts are hidden due to practical reasons.
informatio n object	publication	julkaisu	Any published report, book, web page or similar permanent piece of information that can be unambiguously referenced.
publication	nugget	tiedomuru	An object that is not editable by other people than a dedicated author (group).
substance	topic	aihe	A description of an area of interest. It defines boundaries of a content rather than defines the content itself, which is done by statements. When the information structure is improved, a topic often develops into a question of a knowledge crystal, while a statement develops into an answer of a variable.
priority	objective	tavoite	A desired outcome of a decision. In shared understanding description, it is a topic (or variable) that has value statements attached to it.
substance	risk factor	riskitekijä	

Class	English name	Finnish name	Description
substance	indicator	indikaattori	Piece of information that describes a particular substantive item in a practical and often standard way.
indicator	risk indicator	riski-indikaattori	Indicator about (health) risk or outcome
information object	data	tietoaineisto	
information object	graph	kuvaaja	Graphical representation of a piece of information. Typically is related to an information object with *describes* relation.
work	data work	tietotyö	
work	data use	tiedon käyttö	
substance	priority	prioriteetti	
substance	expense	kustannus	
substance	health impact	terveysvai-kutus	
stakeholder	decision maker	päättäjä	
stakeholder	public officer	virkamies	
stakeholder	assessor	arvioija	
stakeholder	expert	asiantuntija	
stakeholder	citizen	kansalainen	
stakeholder	agent	toimija	
action	task	toimenpide	action to be taken when the option has been selected
action	decision	päätös	action to be taken when the option is yet to be selected. Describes a particular event where a decision maker chooses among defined alternatives. This may also be a part of an assessment under heading Decisions and scenarios.
action	work	työ	continuous actions of the same kind and typically independent of the decision at hand. If the decision changes work routines, the action to make this change happen is called task.
work	prevention	ennaltaeh-käisy	trying to prevent something
work	treatment	hoito	trying to fix something when something has already happened

Class	English name	Finnish name	Description
work	support	tuki	work that aids in the completion of the selected option, in whatever way
method	open policy practice	avoin päätöksen-tekokäytäntö	framework for planning, making, and implementing decisions
method	open assessment	avoin arviointi	method answering this question: How can factual and value information be organised for supporting societal decision making when open participation is allowed?
method	analysis	analyysi	
method	reporting	raportointi	
method	measureme nt	mittaus	
publication	study	tutkimus	
publication	encyclopedi a article	ensyklope-dia-artikkeli	An object that describes a topic rather than answers a specific research question.
publication	lecture	luento	Contains a piece of information that is to be mediated to a defined audience and with a defined learning objective.
method	procedure	toiminta-malli	
method	principle	periaate	a short generic guidance for information work to ensure that the work is done properly. They especially apply to the execution phase.
principle	intentionalit y	tavoitteelli-suus	See Table 3 for explanations.
principle	causality	syysuhteiden kuvaus	
principle	criticism	kritiikki	
principle	permanent resource locations	kohteellisuu s	
principle	openness	avoimuus	
principle	reuse	uusiokäyttö	
principle	use of knowledge crystals	tietokiteiden käyttö	
principle	grouping	ryhmäyty-minen	Facilitation methods are used to promote the participants' feeling of being an important member of a group that has a meaningful purpose.

Class	English name	Finnish name	Description
principle	respect	arvostus	Contributions are systematically documented and their merit evaluated so that each participant receives the respect they deserve based on their contributions.
objective	expense objective	kustannus-tavoite	
process	step	jakso	one of sequential time intervals when a particular kind of work is done in decision support. In the next step, the nature of the work changes.
step	impact assessment	vaikutusa-rviointi	the first step in a decision process. Helps in collecting necessary information for making a decision.
step	decision making	päätöksen-teko	the second step in a decision process. When the decision maker actually chooses between options.
step	implementa tion	toimeen-pano	the third step in a decision process. When the chosen option is put in action.
step	evaluation	evaluointi	the fourth step in a decision process. When the outcomes of the implementation are evaluated.
process	phase	vaihe	one part of a decision work process where focus is on particular issues or methods. Typically phases overlap temporally.
phase	shared under-standing	jaettu ymmärrys	documenting of all relevant views, facts, values, and opinions about a decision situation in such a way that agreements and disagreements can be understood
phase	execution	toteutus	production of necessary information for a decision at hand
phase	evaluation and manage-ment	seuranta ja ohjaus	ensuring that all work related to a decision will be, is, and has been done properly
phase	co-creation	yhteiskehit-täminen	helping people to participate, contribute, and become motivated about the decision work

Relation types

Relations are edges between items (or nodes). A relation I is said to be an inverse of relation R, iff, for all items subject and object, claim "subject R object" is always equal to claim "object I subject".

Table S3-2. Relation types used in open policy ontology.

Class	English name	Finnish name	English inverse	Finnish inverse	Description
relation	participatory link	osallisuus-linkki			The subject is a stakeholder that has a particular role related to an object
relation	operational link	toiminto-linkki			The subject has some kind of practical relation to the object (a fairly wide class)
relation	evaluative link	arvostus-linkki			The subject shows preference or relevance about the object
relation	referential link	viitelinkki			The subject is used as a reference of a kind for the object
relation	argumentative link	argumentaa-tiolinkki			The subject is used as an argument to criticise the object.
relation	causal link	syylinkki			The subject has causal effect on the object (or vice versa in the case of an inverse relation)
relation	property link	ominaisuus-linkki			The object describes a defined property of the subject.
causal link	negative causal link	negatiivinen syylinkki			The subject reduces or diminishes the object.
causal link	positive causal link	positiivinen syylinkki			The subject increases or enhances the object.
negative causal link	decreases	vähentää	is decreased by	vähentyy	
positive causal link	increases	lisää	is increased by	lisääntyy	
negative causal link	worsens	huonontaa	is worsened by	huonontuu	
positive causal link	improves	parantaa	is improved by	parantuu	
negative causal link	prevents	estää	is prevented by	estyy	
positive causal link	enhances	edistää	is enhanced by	edistyy	

Class	English name	Finnish name	English inverse	Finnish inverse	Description
negative causal link	impairs	heikentää	is impaired by	heikentyy	
positive causal link	sustains	ylläpitää	is sustained by	ylläpitäytyy	
causal link	affects	vaikuttaa	is affected by	vaikuttuu	
causal link	indirectly affects	vaikuttaa epäsuorasti	indirectly affected by	vaikuttuu epäsuorasti	
causal link	cause of	syy	caused by	johtuu	Wikidata property P1542
causal link	immediate cause of	välitön syy	immediately caused by	johtuu välittömästi	Wikidata property P1536
causal link	contributin g factor of	vaikuttava tekijä			Wikidata property P1537
participatory link	performs	toteuttaa	performer	toteuttajana	who does a task?
participatory link	decides	päättää	decider	päätäjänä	
participatory link	asks	kysyy	asker	kysyjänä	
participatory link	participates	osallistuu	participant	osallistujana	
participatory link	accepts	hyväksyy	accepted by	hyväksyjänä	
participatory link	develops	kehittää	developed by	kehittäjänä	
participatory link	proposes	ehdottaa	proposed by	ehdottajana	
participatory link	answers	vastaa	answered by	vastaajana	
participatory link	responsible for	vastuussa	responsibility of	vastuullisena	
participatory link	negotiates	neuvottelee	negotiated by	neuvotteli-jana	
participatory link	recommend s	suosittelee	recommende d by	suosittelijana	
participatory link	controls	kontrolloi	controlled by	kontrolloijana	
participatory link	claims	väittää	claimed by	väittäjänä	

Class	English name	Finnish name	English inverse	Finnish inverse	Description
participatory link	owns	omistaa	owned by	omistajana	
participatory link	does	tekee	done by	tekijänä	
participatory link	maintains	ylläpitää	maintained by	ylläpitäjänä	
participatory link	oversees	valvoo	overseen by	valvojana	
operational link	has option	omistaa vaihtoehdon	option for	vaihtoehtona	
operational link	has index	omistaa indeksin	index for	indeksinä	
operational link	tells	kertoo	told by	kertojana	
operational link	describes	kuvaa	described by	kuvaajana	
operational link	maps	kartoittaa	mapped by	kartjoittajana	
operational link	contains data	sisältää dataa	data contained in	data sisältyy	
operational link	data for	on datana	gets data from	saa datansa	
operational link	uses	käyttää	is used by	on käytettävänä	an input (object) for a process (subject)
operational link	produces	tuottaa	is produced by	tuottajana	Object is an output of a process produced by a stakeholder (subject)
operational link	provides	varustaa	is provided by	varustajana	
property link	logical link	looginen linkki			Relations based on logic
property link	set theory link	joukko-oppilinkki			Relations based on set theory
set theory link	part of	osana	has part	sisältää osan	is a part of a bigger entity, e.g. Venus is part of Solar System. Wikidata property P361 (part of) & P527 (has part).
set theory link	context for	kontekstina	has context	omistaa kontekstin	

Class	English name	Finnish name	English inverse	Finnish inverse	Description
set theory link	has subclass	omistaa alajoukon	subclass of	alajoukkona	Wikidata property P279
set theory link	has instance	omistaa instanssin	instance of	instanssina	Object belongs to a set defined by the subject and inherits the properties of the set. Sysnonym for has item, which is depreciated. Wikidata property P31
logical link	opposite	vastakohta			subject is opposite of object, e.g. black is opposite of white. Wikidata property P461; it is its own inverse
logical link	inverse	toisinpäin			a sentence is equal to another sentence where subject and object switch places and has the inverse relation. This is typically needed in preprocessing of insight networks, and it rarely is explicitly shown of graphs. Wikidata property P1696; it is its own inverse
logical link	if - then	jos - niin	if not - then not	jos ei - niin ei	If subject is true, then object is true. Also the negation is possible: if - then not. This links to logical operators and, or, not, equal, exists, for all; but it is not clear how they should be used in an insight network.
operational link	prepares	valmistelee	prepared by	valmistelijana	
operational link	pays	kustantaa	paid by	kustantajana	
operational link	rationale for	perustelee	has rationale	perusteltuu	
operational link	offers	tarjoaa	offered by	tarjoajana	
operational link	executes	suorittaa	executed by	suorittajana	

Class	English name	Finnish name	English inverse	Finnish inverse	Description
operational link	irrelevant to	epärelevantti asiassa			If there is no identified relation (or chain of relations) between a subject and an object, it implies that the subject is irrelevant to the object. However, sometimes people may (falsely) think that it is relevant, and this relation is used to explicate the irrelevance.
evaluative link	finds important	kokee tärkeäksi	is found important	tärkeäksi kokijana	
evaluative link	makes relevant	tekee relevantiksi	is made relevant	relevantiksi tekijänä	if the subject is valid in the given context, then the object is relevant. This typically goes between arguments, from a variable to value statement or from a value statement to a fact statement. This is a synonym of 'valid defend of type relevance'.
evaluative link	makes irrelevant	tekee epärelevan-tiksi	is made irrelevant	epärelevan-tiksi tekijänä	Opposite of 'makes relevant'. Synonym of 'valid attack of type relevance'.
evaluative link	makes redundant	tekee turhaksi	is made redundant	turhaksi tekijänä	Everything that is said in the object is already said in the subject. This depreciates the object because it brings no added value. However, it is kept for archival reasons and to demonstrate that the statement was heard.
evaluative link	has opinion	on mieltä			Subject (typically a stakeholder) supports the object (typically a value or fact statement). This is preferred over 'values' and 'finds important' because it is more generic without loss of meaning.
evaluative link	values	arvostaa	valued by	arvostajana	A stakeholder (subject) gives value or finds an object important. Object may be a topic or statement. Depreciated, use 'has opinion' instead.

Class	English name	Finnish name	English inverse	Finnish inverse	Description
evaluative link	has truthlike-ness	on totuudelli-nen			A subjective probability that subject is true. Object is a numeric value between 0 and 1. Typically this has a qualifier 'according to X' where X is the person or archetype who has assigned the probability.
evaluative link	has preference	mieltymys	preference of	mieltymyk-senä	Subject is better than object in a moral sense.
evaluative link	has popularity	on suosiossa			A measure based on likes given by users.
evaluative link	has objective	omaa tavoitteen	objective of	tavoitteena	
argumentati ve link	agrees	samaa mieltä			
argumentati ve link	disagrees	eri mieltä			
argumentati ve link	comments	kommentoi	commented by	kommentoi-jana	
argumentati ve link	defends	puolustaa	defended by	puolustajana	
argumentati ve link	attacks	hyökkää	attacked by	hyökkääjänä	
argumentati ve link	relevant argument	relevantti argumentti			Argument is relevant in its context.
argumentati ve link	irrelevant argument	epärelevantti argumentti			Argument is irrelevant in its context.
argumentati ve link	joke about	vitsi aiheesta	provokes joke	kirvoittaa vitsin	This relation is used to describe that the subject should not be taken as information, even though it may be relevant. Jokes are allowed because they may help in creating new ideas and perspectives to an issue.

Class	English name	Finnish name	English inverse	Finnish inverse	Description
referential link	topic of	aiheena	has topic	aiheesta	This is used when the object is a publication and the subject is a (broad) topic rather than a statement. In such situations, it is not meaningful to back up the subject with references. Useful in describing the contents of a publication, or identifying relevant literature for a topic.
referential link	discussed in	kerrotaan	discusses	kertoo	
referential link	reference for	viitteenä	has reference	viite	Subject is a reference that backs up statements presented in the object. Used in the same way as references in scientific literature are used.
referential link	states	väittää	stated in	väitetään kohteessa	Describes the source of a statement; may also refer to a person.
referential link	tag for	täginä	has tag	omistaa tägin	Subject is a keyword, type, or class for object. Used in classifications.
referential link	category for	kategoriana	has category	kuuluu kategoriaan	
referential link	associates with	liittyy			Subject is associated with object in some undefined way. This is a weak relation and does not affect the outcomes of inferences, but it may be useful to remind users that an association exists and it should be clarified more precisely. This is its own inverse.
referential link	answers question	vastaa kysymyk-seen	has answer	vastaus	Used between a statement (answer) and a topic (question). In knowledge crystals, the relation is embedded in the object structure.
irrelevant argument	irrelevant comment	epärelevantti kommentti			Inverses are not needed, because the relation is always tied with an argument (the subject).
irrelevant argument	irrelevant attack	epärelevantti hyökkäys			
irrelevant argument	irrelevant defense	epärelevantti puolustus			

Class	English name	Finnish name	English inverse	Finnish inverse	Description
relevant argument	relevant comment	relevantti kommentti			
relevant argument	relevant attack	relevantti hyökkäys			
relevant argument	relevant defense	relevantti puolustus			
property link	evaluative property	arviointi-ominaisuus			characteristic of a product or work that tells whether it is fit for its purpose. Especially used for assessments and assessment work.
evaluative property	property of decision support	päätöstuen ominaisuus			What makes an assessment or decision support process fit for its purpose?
evaluative property	setting of assessment	arvioinnin kattavuus			See Table 5.
setting of assessment	impacts	vaikutukset			
setting of assessment	causes	syyt			
setting of assessment	problem owner	asianomis-taja			
setting of assessment	target users	kohderyhmä			
setting of assessment	interaction	vuorovai-kutus			
interaction	dimension of openness	avoimuuden ulottuvuus			See Table 6.
dimension of openness	scope of participatio n	osallistumi-sen avoimuus			
dimension of openness	access to information	tiedon avoimuus			
dimension of openness	timing of openness	osallistumi-sen ajoitus			
dimension of openness	scope of contributio n	osallistumi-sen kattavuus			

Class	English name	Finnish name	English inverse	Finnish inverse	Description
dimension of openness	impact of contribution	osallistumisen vaikutus			
interaction	category of interaction	vuorovaikutuksen luokka			See Table 2. How does assessment interact with the intended use of its results? Possible values: isolated (eristetty), informing (tiedottava), participatory (osallistava), joint (yhteistyöhakuinen), shared (jaettu).
property of decision support	quality of content	sisällön laatu			See Table 4.
quality of content	informativeness	tarkkuus			
quality of content	calibration	harhattomuus			
quality of content	coherence	sisäinen yhdenmukaisuus			
property of decision support	applicability	sovellettavuus			
applicability	relevance	merkityksellisyys			
applicability	availability	saatavuus			
applicability	usability	käytettävyys			
applicability	acceptability	hyväksyttävyys			
property of decision support	efficiency	tehokkuus			
efficiency	intra-assessment efficiency	sisäinen tehokkuus			
efficiency	inter-assessment efficiency	ulkoinen tehokkuus			

Appendix S4: Workspace tools: OpasnetUtils package and Opasnet Base

Ovariable

Ovariable is an object class that is used in R to operationalise knowledge crystals. In essence, impact assessment models are built using ovariables as the main tool to organise, analyse, and synthesise data and causal relations between items. The purpose of ovariables is to offer a standardised, generalised, and modular solution to modelling. Standardised means that all ovariables have the same overall structure, and this makes it possible to develop generalised functions and processes to manipulate them. Modular structure of a model makes it possible to change pieces within the model without braking the overall structure of functionality. For example, it is possible to take an existing health impact model, replace the ovariable that estimates the exposure of the target population with a new one, and produce results that are otherwise comparable to the previous results but differ based on exposure.

What is the structure of an ovariable such that

- it complies with the requirements of variable and
- it is able to implement probabilistic descriptions of multidimensional variables and
- it is able to implement different scenarios?

An ovariable contains the current best answer in a machine-readable format (including uncertainties when relevant) to the question asked by the respective knowledge crystal. In addition, it contains the information needed to derive the current best answer. The respective knowledge crystal typically has an own page at Opasnet, and the code to produce the ovariable is located on that page under subheading Calculations.

It is useful to clarify terms here. *Answer* is the overall answer to the question asked (including an evaluated ovariable), and it is the reason for producing the knowledge crystal page in the first place. Answer is typically located near the top of the page to emphasise its importance. An answer may contain text, tables, or graphs on the web page. It typically also contains an R code for evaluating the respective ovariable. *Output* is the key part (technically a slot) of the answer within an ovariable and contains the details of what the reader wants to know about the answer. All other parts of the ovariable are needed to produce the output or understand its meaning. Finally, *Result* is the key column of the Output table (technically a data frame) and contains the actual numerical values for the answer.

Slots

The ovariable is a class S4 object defined by OpasnetUtils in R software system. An ovariable has the following separate *slots* that can be accessed using X@slot (where X is the name of the ovariable):

@name

- Name of <self> (the ovariable object) is useful since R's S4 classes doesn't support self reference. It is used to identify relevant data structures as well as to set up hooks for modifiers such as scenario adjustments.

@output

- The current best answer to the question asked.
- A single data frame (a 2D table type in R)
- Not defined until <self> is evaluated.
- Possible types of columns:
    - *Result* is the column that contains the actual values of the answer to the question of the respective knowledge crystal. There is always a result column, but its name may vary; it is of type <self>Result.
    - *Indices* are columns that define or restrict the Result in some way. For example, the Result can be given separately for males and females, and this is expressed by an index column *Sex*, which contains locations *Male* and *Female*. So, the Result contains (at least) one row for males and one for females. If there are several indices, the number of rows is typically the product of numbers of locations in each index. Consequently, the output may become very large with several indices.
    - *Iter* is a special kind of index used in Monte Carlo simulations. Iter contains the number of the iteration. In Monte Carlo, the model is typically run 1000 or 10000 times.
    - *Unit* contains the unit of the Result. It may be the same for all rows, but it may also vary from one row to another. Unit is not an index.
    - Other, non-index columns can exist. Typically, they are information that were used for some purpose during the evolution of the ovariable, but they may be unimportant in the current ovariable if they have been inherited from parent ovariables. Due to these other columns, the output may sometimes be rather wide.

@data

- A single data frame that defines <self> as such.
- *data* slot contains data about direct measurements or estimates of the output. Typically, when data is used, the output can be directly derived from the information given, with possibly some manipulations such as dropping out unnecessary rows or interpreting given ranges or textual expressions as probability distributions.
- Probability distributions are interpreted by *OpasnetUtils/Interpret*.

@marginal

- A logical vector that indicates full marginal indices (and not parts of joint distributions, result columns, or units or other row-specific descriptions) of output.

@formula

- A function that defines <self> using objects from dependencies as inputs.
- Returns either a data frame or an ovariable, which is then used as the output of the ovariable.
- Formula and dependencies slots are always used together. They estimate the answer indirectly in cases when there is knowledge about how this variable depends on the results of other variables (called parents). The slot dependencies is a table of parent variables and their identifiers, and formula is a function that takes the outputs of those parents, applies the defined code to them, and in this way produces the output for this variable.

@dependencies

- A data frame that contains names and tokens or identifiers for model runs of variables required for <self> evaluation (list of causal parents). The following columns may be used:
    - Name: name of an ovariable or a constant found in the global environment (.GlobalEnv).
    - Key: the run key (typically a 16-character alphanumeric string) of a model run that is stored to Opasnet server. Key to be used in objects.get() function to fetch the dependent object.
    - Ident: Page identifier and rcode name to be used in objects.latest() function where the newest run contains the dependent object. Syntax: "Op_en6007/answer".
    - Also other columns are allowed (e.g. Description), and they may contain additional information about parents.
- Dependencies is a way of enabling references in ovariables by using function OpasnetUtils/ComputeDependencies. It creates variables in .GlobalEnv environment so that they are available to expressions in formula.
- Dependent ovariables are fetched and evaluated (only once by default) upon <self> evaluation.

@ddata

- A string containing an Opasnet identifier e.g. "Op_en1000". May also contain a subset specification e.g. "Op_en1000/dataset".
- This identifier is used to download data from the Opasnet database for the data slot (by default, only if empty) upon <self> evaluation.
- By default, the data defined by ddata is downloaded when an ovariable is created. However, it is also possible to create and save an ovariable in such a way that the data is downloaded only when the ovariable is evaluated.

@meta

- A list of descriptive information of the object. Typical information include date created, username of the creator, page identifier for the Opasnet page with the ovariable code, and identifier of the model run where the object was created.
- Other meta information can be added manually.

OpasnetUtils and operations with ovariables

OpasnetUtils is an R package found in CRAN repository (cran.r-project.org). It contains tools for open assessment and modelling at Opasnet, especially for utilising ovariables as modelled representations of knowledge crystals. Typically, ovariables are defined at Opasnet pages, and their data and evaluated output are stored to Opasnet server. There are also special user interface tools to enable user inputs before an R code is run on an Opasnet page; for further instructions, see http://en.opasnet.org/w/R-tools. However, ovariables can be used independently for building modular assessment models without any connection to Opasnet.

The example code shows some of the most important functionalities. Each operation is followed by an explanatory comment after # character.

```
install.packages("OpasnetUtils") # Install the package OpasnetUtils. This is done
only once per computer.

library(OpasnetUtils) # Open the package. This is done once per R session.

objects.latest("Op_en4004", code_name="conc_mehg") # Fetch ovariables stored by
code conc_mehg at Opasnet page Mercury concentrations in fish in Finland (with
identifier 4004)

conc_mehg <- EvalOutput(conc_mehg) # Evaluate the output of ovariable conc_mehg
(methyl mercury concentrations in fish) that was just fetched.

dat <- opbase.data("Op_en4004", subset="Kerty database") # Download data from Kerty
database on the same page and put that to data.frame dat

a <- Ovariable("a", data=data.frame(Fish=c("Herring","Salmon"), Result=c(1,3))) #
Define ovariable for scaling salmon results with factor 3.

mehg_scaled <- conc_mehg * a # Multiply methyl mercury concentrations by the
scaling factor.
```

An ovariable is well defined when there is enough data, code or links to evaluate the output. Ovariables often have upstream dependencies whose output affect the output of the ovariable at hand. Therefore, ovariables are usually stored in a well defined but unevaluated format (i.e. without output). This makes it possible to use the same ovariable in different contexts, and the output varies depending on the upstream dependencies. On the other hand, it is possible to store all evaluated ovariables of a whole assessment model. This makes it possible to archive all details of a certain model version for future scrutiny.

Ovariables have an efficient index handling, which makes it possible to do arithmetic operations such as sums and products in a very simple way with ovariables. The basic idea is that if the outputs of two ovariables have two columns by the same name, they are automatically merged (or joined, using the SQL vocabulary) so that rows are merged iff they have the same location values in those two columns. The same principle applies to all pairs of columns by the same name. After the merge, the arithmetic operation is performed, row by row, to the Result columns of each ovariable. This results in an intuitive handling of outputs using a short and straightforward code.

Recursion is another important property of ovariables. When an ovariable is evaluated, a code checks whether it has upstream dependencies. If it does, those ovariables are fetched and evaluated first, and recursively the dependencies of those ovariables are fetched also, until all dependencies have been evaluated. Case-specific adjustments can be done to this recursion by fetching some upstream ovariables before the first ovariable is evaluated; if an upstream ovariable exists already in the global environment, the existing object is used and the respective stored object is not fetched (dependencies are only fetched if they do not already exist; this is to avoid unnecessary computation).

Decisions and other upstream commands

The general idea of ovariables is such that their code should not be modified to match a specific model but rather define the knowledge crystal in question as extensively as possible under it's scope. In other words, it should answer its question in a reusable way so that the question and answer would be useful in many different situations. (Of course, this should be kept in mind already when the question is defined.) To match the scope of specific models, ovariables can be modified without changing the ovariable code by supplying commands upstream. A typical decision command is to make a new decision index with two scenarios, "business as usual" and "policy" and use the original ovariable result for business as usual and adjust the result for the policy e.g. by adding or multiplying it by a constant reflecting the impact of the policy on the ovariable. Such adjustments can be done on the assessment level without a need to change the ovariable definition in any way.

Evaluating a latent ovariable triggers first the evaluation of its unevaluated parent ovariables (listed in dependencies) since their results are needed to evaluate the child. This chain of evaluation calls forms a recursion tree in which each upstream variable is evaluated exactly once (cyclical dependencies are not allowed). Decision commands about upstream variables are checked and applied upon their evaluation and then propagated downstream to the first variable being evaluated. For example, decisions in decision analysis can be supplied this way:

1. pick an endpoint ovariable
2. make decision variables for any upstream ovariables (this means that you create new scenarios with particular deviations from the actual or business-as-usual answer of that ovariable)
3. evaluate endpoint ovariable
4. optimize between options defined in decisions.

Other commands include: collapse of marginal columns by sums, means or sampling to reduce data size; and passing input from model level without redefining a whole ovariable.

Opasnet Base

Opasnet Base is a storage database for all kinds of data needed in open assessments. It may contain parameter values for models, which are typically shown as small tables on knowledge crystal pages, from which they are automatically stored to the database. It may also contain large dataset such as research datasets or population datasets of thousands or even millions of rows, and they are uploaded to the database using an importer interface. Each table has its own structure and may or may not share

column names with other tables; however, if a table is directly used as data slot for an ovariable, it must have a Result column.

Technically, Opasnet Base is a noSQL database using MongoDB software. Metadata of the tables is stored in a MySQL database. This structure offers the speed, searchability, and structural flexibility that a large amount of non-standard data requires. The database also offers version control, as old versions of a data table are kept in the database when new data is uploaded.

The database also contains data about model runs that have been performed at Opasnet, if objects were stored during that model run. This makes it possible to fetch objects produced by a particular code on a particular knowledge crystal page. Typically the newest version is fetched, but information about the old versions are kept as well. The objects stored are not located in MongoDB but on server files that can be accessed with a key. It is also possible to save objects in a non-public way so that the key is not stored in the database and is only given to the person who ran the code. Due to disc storage reasons, Opasnet does not guarantee that stored objects will be kept permanently; therefore, it is a good practice to store final assessment runs with all objects to another location for permanent archival.

There are several ways to access database content.

- If the data is on an Opasnet page, simply go to that page, e.g. http://en.opasnet.org/w/Mercury_concentrations_in_fish_in_Finland#Data
- Use a link to the Opasnet Base interface, e.g. http://en.opasnet.org/w/Special:Opasnet_Base?id=op_en4004.mercury_in_baltic_herring
- Use a function in R: dat <- opbase.data("Op_en4004", subset="Mercury in Baltic herring")
- Use a function in R for stored objects: objects.latest("Op_en4004", code_name="conc_mehg")

For further instructions, see http://en.opasnet.org/w/Opasnet_Base_UI for user interface and http://en.opasnet.org/w/Table2Base for the wiki interface of small tables.

Appendix S5: Tools to help in shared understanding

There are lots of software and platforms to support decision making. Some of them have been listed here. The focus is on open source software solutions when available. Many examples come from Finland, as we have practical experience about them. The list aims to cover different functionalities and show examples rather than give an exhaustive list of all possibilities; such lists may be found from Wikipedia, e.g. https://en.wikipedia.org/wiki/Comparison_of_project_management_software. All links were accessed 1 Feb 2020.

Table S5-1. Useful functionalities and software in open policy practice.

Item	Functionality or process phase	Tool or software
**Decision process**	Information-based decision support	There is no single tool covering the whole decision process. Development work is needed. An interesting pilot software is being developed by the city of Helsinki for comprehensively managing and evaluating their ambitious Climate Watch and its impacts.
	Initiative	Several websites for launching, editing, and signing citizen initiatives at municipality or national level: Kansalaisaloite (Citizen Initiative), Nuortenideat (Ideas of the Young), Kuntalaisaloite (Municipality Initiatives). Similar tools could be used also for initiatives launched by Members of Parliament or the Government.
**Substance**	Content management	Diary systems, file and content management systems. Lots of individual solutions, mostly proprietary. VAHVA project by the Finnish Government will provide knowledge and tools for content management.
	Research data and analyses	AVAA, IDA, Fairdata and other data management tools help in managing research data from an original study to archival. Avoin data (open data in Finland), platform for publishing open data. Findicator: indicators from all sectors of the society. Datahub for open data sharing. Tools for separate analysis tasks are numerous, e.g. QGIS for geographical data. Several research fields have their own research and article databases, such as ArXiv.org (articles about physics, mathematics and other fields). Several biological databases.
	Public discussion, argumentation, statements	Otakantaa, Facebook, Twitter, blogs, and other social media forums for discussion. Websites for fact checking: Factbar, Fullfact, Need to know project for fact checking. Agoravoting is an open voting system. Lausuntopalvelu collects statements from the public and organisations related to planned legislation and Government programs in Finland. Swarm AI for collective intelligence
	News	News feeds (open source) CommaFeed, Tiny Tiny RSS. Semantic, automated information searches, e.g. Leiki.

Item	Functionality or process phase	Tool or software
	Description and assessment of decision situations and relevant causal connections	Opasnet for performing Open assessments and impact assessments. Knowledge crystals as integral parts of models and assessments. Simantics System Dynamics in semantic models. Jupyter notebooks for collaborative model development. Wikidata, Wikipedia as storages of structured data and information.
	Laws and regulations	Semantic Finlex contains the whole Finnish legislation and e.g. the decisions by the Supreme Court in a semantic structure.
**Methods**	Preparation of documents, co-creation, real-time co-editing	Several co-editing tools, e.g. Hackpad, MS Office365, Google Docs, Etherpad, Dropbox Paper, MediaWiki and Git. These tools enable the opening of the planning and writing phase of a decision. E.g. the climate strategy of Helsinki was co-created online with Google Docs and Sheets in 2018.
	Development and spreading good practices	InnoVillage helps to develop practices faster, when everyone's guidance is available online and can be commented.
	Organising systems for information and discussions	Decentralised social networking protocol Activitypub<br><br>Tools: Full Fact automated fact checking Compendium. Vocabularies and semantic tools: Resourse Description Framework (RDF), Finto (Finnish Thesaurus and Ontology Service), AIF-RDF Ontology using Conceptual Graphics User Interface COGUI. These act as a basis for organising, condensing and spreading knowledge.
	Information design, visualisations	Interactive and static visualisations from complex data. Shiny, Diagrammer, Gapminder, Lucify Plotly Cytoscape
**Work**	Work processes in decision making, research etc: follow-up, documentation	Ahjo decision repository and Openahjo interface document and retrieve decisions that have been done in the city of Helsinki. Git enables reporting of both research and decision processes. There are several new platforms for improving science, such as Open Science Framework for facilitating open collaboration in research. Omidyar Network is a philantropic investment firm supporting e.g. governance and citizen engagement.
	Co-creation, experiments, crowdsourcing	Kokeilun paikka promotes experiments when applicable information is needed but not available. Sociocracy 3.0 provides learning material and principles for open collaboration in organisations of any size.

Item	Functionality or process phase	Tool or software
	Project management	There are lots of project management software, mainly targeted for enterprise use but somewhat applicable in decision making or research. Some examples: OpenProject, Project Management Body of Knowledge, Comparison of project management software, Fingertip.
**Stake-holders**	Expert services	ResearchGate Solved and other expert networks.
